# Rheological Insight into the 3D Printability of Carboxymethyl Cellulose-Based Hydrogels

**DOI:** 10.3390/gels11040259

**Published:** 2025-04-01

**Authors:** Itziar Insua, Oliver Etzold, Itxaso Calafel, Robert Aguirresarobe, Marcelo Calderón, Mercedes Fernández

**Affiliations:** 1 POLYMAT and Department of Advanced Polymers and Materials: Physics, Chemistry and Technology, Faculty of Chemistry, University of the Basque Country UPV/EHU, Paseo Manuel de Lardizabal, 3, 20018 Donostia-San Sebastián, Spain; itziar.insua@ehu.eus (I.I.); itxaso.calafel@ehu.eus (I.C.); 2 POLYMAT and Applied Chemistry Department, Faculty of Chemistry, University of the Basque Country, UPV/EHU, Paseo Manuel de Lardizabal, 3, 20018 Donostia-San Sebastián, Spainmarcelo.calderon@polymat.eu (M.C.); 3 IKERBASQUE, Basque Foundation for Science, Plaza Euskadi 5, 48009 Bilbao, Spain

**Keywords:** hydrogels, 3D printing, yield stress, viscoelasticity, FT and SPP rheology

## Abstract

Direct Ink Writing (DIW) is an advanced additive manufacturing 3D-printing technique with significant potential for producing hydrogels in biomedical and engineering applications. This study presents a comprehensive rheological analysis of the yielding and recovery properties critical for ensuring the printability of carboxymethyl cellulose (CMC)-based hydrogels incorporating atenolol, an antihypertensive agent, as the active ingredient. The viscoelastic properties under shear conditions were examined using Large Amplitude Oscillatory Shear (LAOS) testing. To obtain both qualitative and quantitative insight into hydrogel dynamics, Lissajous-Bowditch plots and Fourier Transform (FT) coefficients were analyzed. The evaluation of stress signal anharmonicity and the decomposition of stress into its elastic and viscous components allowed for distinguishing structural evolution under flow among the tested hydrogels. Additionally, the analysis of the Sequence of Physical Processes (SPP) during each deformation cycle provided deeper insight into oscillatory yielding behavior, emphasizing the role of elastic strains in determining printability. Overall, the study offers valuable understanding of the nonlinear viscoelastic behavior of CMC-based hydrogels, providing a framework for optimizing hydrogel formulations in DIW applications.

## 1. Introduction

The rheology of complex fluids tailored for direct ink writing (DIW) has been a prominent area of research over the past decade due to the potential versatility of this additive manufacturing technique across diverse fields, including material science and biomedicine [1,2,3,4,5,6]. DIW allows the precise deposition of materials in 3D patterns, enabling the fabrication of complex structures from a wide range of materials. The process critically depends on the interplay between material structural properties and the processing conditions. Therefore, a deep understanding of the rheological behavior of printable materials is critical for advancing DIW and expanding its range of applications. While the connection between rheology and DIW printability is widely recognized and extensively documented in the literature [7,8,9,10,11,12,13,14], defining this precise relationship quantitatively remains difficult, due to the wide variety of soft materials used and the diversity of rheological testing methodologies. This study aims to contribute to this understanding by investigating the rheological parameters that define the optimal printability of yield-stress hydrogels, specifically analyzing formulations based on carboxymethyl cellulose (CMC) hydrogels designed for atenolol administration.

Overall, 3D-printed CMC-based hydrogels have found clinical applications due to their excellent biocompatibility, easy modifiability, and high potential for drug encapsulation, making them attractive for biomedical applications. However, CMC alone often suffers from poor mechanical strength due to the formation of intramolecular rather than intermolecular cross-links [15,16]. To overcome this limitation, we considered polyethylene glycol (PEG) and glycerol (GLY) as candidates to achieve good printability without the need for post-treatment. The inclusion of atenolol, an antihypertensive agent, provides an opportunity to evaluate how the drug affects the rheological properties and printability of these hydrogels.

For successful printing, the “yield-stress” ink must behave as a liquid during extrusion through the nozzle and transition into a solid upon deposition to maintain its shape and support subsequent layers. In yield-stress materials, this solidification step occurs due to a reversible transition from a solid to a liquid state above a critical stress threshold. Thus, the rheological investigation will center on the following properties: (1) flowability—the ability of the hydrogel to flow smoothly through the nozzle without clogging or breaking; (2) yield strength—ensuring sufficient resistance to deformation under the stresses encountered during printing; and (3) recoverability—the ability of the hydrogel to regain its structural integrity and modulus after extrusion, preventing sinking or collapse post-printing [10]. Quantifying these aspects of printability through well-designed rheological experiments presents significant challenges. To address this, our study emphasizes the exploration of oscillatory shear data obtained under large-amplitude oscillatory shear (LAOS), an experimental approach that has demonstrated strong potential in advancing the understanding of the rheology of yield-stress fluids [11,17,18,19,20,21,22].

LAOS experiments enable precise control over the oscillation frequency, determining the time scale, as well as the amplitude of the applied shear deformation. The response in the LAOS region, where large and fast deformations are applied, cannot be treated only by *G′* and *G″* [23,24,25]. The resulting non-sinusoidal stress is typically examined using Fourier-transform rheology (FTR), which converts periodic signals from time to frequency domain. As a result, the nonlinear stress responses are described by the fundamental applied frequency and its higher harmonics. The pioneering work of Wilhelm et al. [25,26,27,28,29,30,31] has advanced FTR-based analysis methodologies, particularly by explaining the use of the ratio of the intensity of the third harmonic to the intensity of the first harmonic [32,33,34]. This ratio, proportional to the square of the applied strain amplitude, serves as a key metric for quantifying nonlinear material behavior. Beyond FTR analysis, Lissajous-Bowditch (L-B) curves, typically representing stress vs. strain and stress vs. strain rate, capture nonlinearities from a geometrical perspective, facilitating a meaningful interpretation of the material’s elastic and viscous components. A notable contribution comes from Cho et al. [35], who introduced a stress decomposition framework based on even and odd functions of the strain and strain rate that enables the determination of elastic and viscous stresses. The Cho’ stress decomposition in terms of the Chebyshev coefficients (*e*_n_ and *ν*_n_) found support from Ewoldt et al. [25,36,37], who proposed physical interpretations for the first two terms in the expansions of Cho’s elastic and viscous stress: the positive third-order elastic coefficient (e_3_ > 0) was interpreted as a stiffening nonlinearity, whereas the positive third Chebyshev coefficient in the description of the viscous stress (ν_3_) was interpreted as thickening nonlinearity. An alternative perspective on L-B curves was offered by Roger et al. [38,39,40,41,42], who emphasized the significance of locally linear regions that could be easily interpreted as sequences of elastic and viscous processes, (Sequence of Physical Processes, SPP, framework), thereby linking the nonlinear LAOS response to fundamental material properties typically obtained from traditional linear viscoelasticity and steady-state viscometrical flow measurement.

Although there is no consensus on the methodologies or formalism to extract unambiguous physical interpretations from LAOS data, these theoretical frameworks enable the evaluation of the stress–strain nonlinear relationship, which is crucial for analyzing structural arrangements occurring over a wide range of frequencies and deformations. Most interestingly, nonlinear viscoelastic parameters have been reported to be promising indicators of the flow and recoverability properties expected in 3D-printable materials. García-Tuñon et al. reported the behavior of a different set of carbon-based formulations, comprising graphene (Gr), graphene oxide (GO), and carbon nanotubes (CNTs), examined by FTR [12] and an SPP framework [11]. This made it possible to identify the occurrence of nonlinearities by examining the appearance of higher (odd) harmonics in the output stress signals obtained during the strain sweep experiments and to select rheological metrics to quantify flowability, recoverability, and material strength. The quantitative framework of the SPP was also investigated on anodic slurry systems containing graphene (Gr), carbon black (CB), and CMC, revealing the existence of intra-cycle rheological transition associated with a two-step yielding behavior represented by double deltoids [43]. Hyeong Yong Song et al. interpreted the three physical processes (recovery, elastic softening, and yielding) experienced within an oscillation cycle in terms of the printability evaluation of liquid Ga-oxide amalgams [44]. These insights from LAOS data provide a thorough understanding of the yielding response, which will be explored in this study as a potential key factor in the 3D-printing process.

The organization of this paper aimed at providing a comprehensive analysis of the yield-stress rheological behavior and printability of the hydrogels. The discussion of the results is structured as follows: Section 2.1 details the formulation of the CMC-based hydrogels designed for atenolol administration. Section 2.2 focuses on the printability of the investigated formulations under a specific printing configuration. Section 2.3 includes the basic rheological characterization, and Section 2.4 is devoted to the interpretation of nonlinear viscoelasticity by oscillatory LAOS data. The LAOS analysis incorporates Fourier-transform rheology (FTR), stress decomposition (FTR-SD), and the Sequence of Physical Processes (SPP) approach. In summary, this study investigates the correlation between nonlinear viscoelasticity, structural reorganization, and dynamic interactions within the hydrogel architecture during deformation. These factors, in turn, influence the printability of the hydrogels. By elucidating these relationships, we aim to develop a comprehensive framework for the rational design of advanced materials, emphasizing the interplay between processing, structure, and rheology.

## 2. Results and Discussion

### 2.1. CMC-Based Hydrogels

CMC was used in this work as the primary structural and functional component of 3D-printed hydrogels for the encapsulation of atenolol, an antihypertensive drug. The remarkable properties of CMC, which include acceptable mechanical properties, excellent hydrogel-forming capacity, biocompatibility, and biodegradability are used to explore its potential as a 3D-printed system for use in personalized medicine.

**Figure 1:**
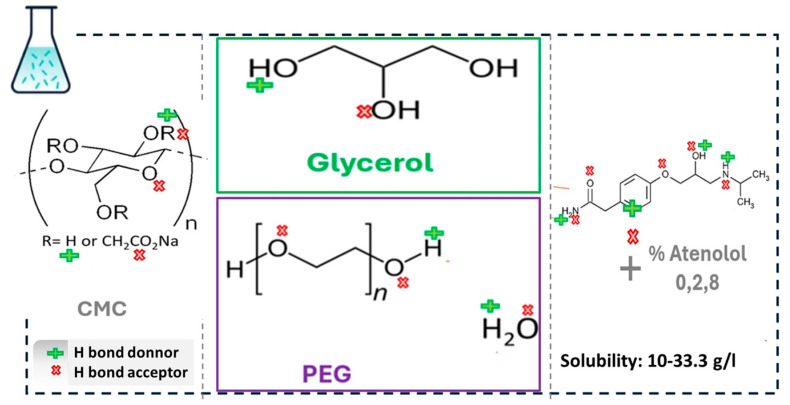
Design formulation of the CMC-based hydrogels. Samples containing polyethylene glycol in water (CMC-PEG) are expected to improve interactions in the dispersed phase, while samples containing glycerol instead of water (CMC-GLY) are expected to increase the viscosity of the continuous phase. The improved intermolecular interactions will result in highly structured hydrogels.

Carboxymethyl cellulose (CMC) is a chemically modified cellulose derivative in which hydroxyl groups in the glucopyranose chains are substituted with carboxymethyl groups (-CH_2_COOH). The β-1,4-glycosidic bonds linking the polysaccharide repeating units (Figure 1) result in an anionic, semiflexible, and water-soluble polyelectrolyte. To enhance the mechanical strength of CMC hydrogels, two sets of formulations were developed by incorporating polyethylene glycol (PEG) and glycerol (GLY). PEG is a common hydrophilic, biocompatible material degradable by oxidative decomposition under biological conditions [45], and GLY is a nontoxic biodegradable solvent commonly used in the synthesis of pharmaceutically active ingredients [46].

These CMC–PEG and CMC–GLY formulations are rich in carboxyl and hydroxyl functional groups (Figure 1), which can develop a structured network primarily governed by intermolecular forces, including hydrophobic, electrostatic, steric, and other non-covalent interactions, such as physical entanglement. The fluidity and reversibility of the hydrogel network will be governed by the balance of these complex interactions. CMC has a strong tendency to form intermolecular hydrogen bonds with polymers containing highly electronegative groups. Polymers such as PEG, which contain electronegative oxygen, are effective proton acceptors due to their functional groups. Consequently, the dominant interaction in the CMC-PEG system is hydrogen bonding between the functional groups of PEG and CMC. In contrast, glycerol acts as a plasticizer by dynamically modifying CMC’s intermolecular and intramolecular hydrogen bonds. Rich in hydroxyl groups, glycerol interacts with CMC’s functional groups, enhancing polymer chain mobility. While PEG strengthens intermolecular interactions within the carboxymethyl cellulose network, glycerol increases viscosity but also functions as a lubricant that makes the system more flexible, improving the flow.

As a result of these interactions, cohesive hydrogel structures were obtained for CMC-PEG and CMC-GLY formulations that were adjusted to achieve the reference hydrogel with an elastic modulus of approximately 100 Pa. These CMC-based hydrogels were used to encapsulate atenolol at concentrations of 2 wt% and 8 wt%. Details are provided in Section 4.

The influence of the plasticizer and the interactions between polymers may potentially affect the dispersion of atenolol. Atenolol is a β1-adrenoceptor blocker commonly used alone or in combination with other medications to treat high blood pressure. Its structure includes an aromatic group with various substituents and an alkanolamine side-chain terminating in a secondary amino group, as shown in Figure 1. Atenolol is freely soluble in water, with a reported solubility of 13.5–33 mg/mL. The formation of hydrogen bonding and electrostatic interactions due to the secondary amine group in the solubilized atenolol are expected to increase intermolecular interactions. In the case where atenolol remains non-solubilized, the particles were dispersed within the hydrogel network, forming a multi-component but stable hydrogel system.

Specifically, the 8 wt% atenolol–CMC-based hydrogels were formulated to match a typical preparation dose for adult patients. Conventionally, atenolol preparations are available as 25 mg, 50 mg, and 100 mg oral tablets; 0.5 mg/mL intravenous injections; and 2–5 mg/mL oral suspensions [47]. Our 3D-printed hydrogels offer an alternative to these traditional formulations and stand among other innovative approaches explored for atenolol administration (transdermal drug delivery systems [48], oral hydrogels [49,50], eutectogel-based systems [51], and lipid based-solid lipid dispersions [52]). The standard starting dose for adults is 50 mg once daily, which can be increased to 100 mg daily after a few weeks if necessary. Therefore, the CMC-PEG-A8 or CMC-GLY-A8 formulations, with a tablet weight of 625 mg, could effectively deliver the 50 mg dose of atenolol, matching dosing recommendations. The simple preparation process for these formulations, outlined in the Materials and Methods section, offers a scalable and adaptable strategy for incorporating other drugs into the hydrogel system.

### 2.2. Direct Ink Writing of CMC-Based Hydrogel: Printing-Strength Characterization

**Figure 2:**
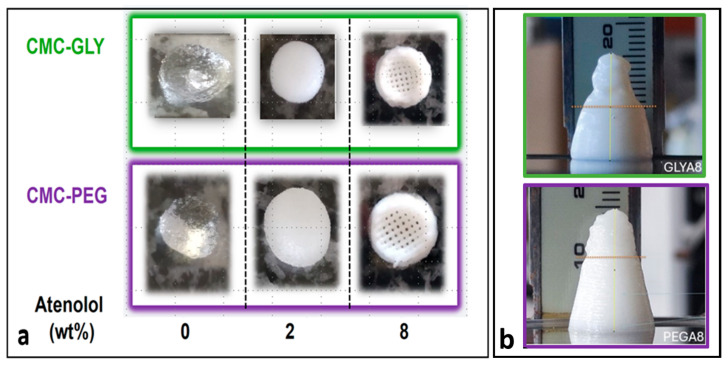
(**a**) 3D-printed structures of CMC-based hydrogels’ with formulation of CMC-GLY and CMC-PEG, containing atenolol at concentrations of 0, 2, and 8 wt%. The resolution of the spacing between filaments increases for the higher content of atenolol. CMC-PEG hydrogels obtained better resolution compared to CMC-GLY with the same content of atenolol. (**b**) Adapted slump cone method for determination of the consistency of shape related to printing strength**,** 15 mm DIW slump cones for CMC-GLY-A8 and CMC-PEG-A8 hydrogels.

All the carboxymethyl cellulose-based formulations prepared in this work were “extrudable”. They were able to flow easily through the nozzle tip and recovered to varying degrees when deposited on the printer platform in the shape of a tablet. However, as can be seen in Figure 2a, the quality of the prints depends on the formulation and, in particular, on the atenolol content. Tablets produced from formulations with 8 wt% atenolol showed better resolution or shape retention than those containing only 2 wt% or no atenolol.

The shape fidelity of DIW-CMC hydrogel-based formulations decreases with decreasing atenolol concentration. This ability to maintain printing resolution during filament deposition is rheologically dependent on the thixotropic character of the sample, because it is required a reasonable “high recoverable yield stress” after the previous extrusion process at high shear rates. However, directly correlating rheology with printability is complex given the different rheological definitions of yield stress and its dependence on material flow history, as we will discuss in the next sections. In this sense, it seems appropriate to attempt to quantify this property during the printing process itself. We focus on the particularly interesting and relatively simple method proposed by Liu et al. [7] that uses a printed slump cone geometry specifically designed to test the strength property of the printed object by analyzing the potential gravitational defects observed post-printing. The slump cone test is commonly used to quantify the consistency of concrete, both in the laboratory and in the field [53]. The size and shape of the slump cone allow us to make a quick determination of how well the concrete will hold its shape based on rheological analysis of the deformation of the cone. Inspired by this methodology, Liu et al. [7] demonstrated that the ability of the slump cone to retain its printed shape provided a measure of the strength of the materials used in the DIW. For uncured filled PDMS materials, they found that the rheological yield stress, measured from creep and oscillatory strain amplitude sweeps, provided a theoretical upper bound of the height of the cone that could be printed, assuming the material rapidly rebuilds stress after high shear rates in the nozzle of the printing head.

According to Liu et al. [7], the height of the undeformed cone allows us to calculate the experimental yield stress experienced by the DIW material using the following equation:

(1) $$\sigma_{\;y}^{p}\approx g{\rho h}_{undeformed}$$

where g = 9.8 m/s^2^ is the gravitational constant, and ρ = 1 g/cm^3^ is the approximate density of the hydrogels’ formulations.

Materials with low yield stress tend to flow after printing, causing the printed tablets to resemble a puddle, as seen for the formulations CMC-PEG-A0, CMC-PEG-A2, CMC-GLY-A0, and CMC-GLY-A2 in Figure 2a. This represents the extreme case where the printing strength is effectively zero. In contrast, a perfect cone, with predicted dimensions and no yielded material at the bottom part, indicates the material’s yield stress exceeds the gravitational force exerted on it. Therefore, materials with high yield strength can maintain their 3D-printed shape effectively, as a larger *h_undeformed_* value indicates higher yield strength and, consequently, better performance in DIW applications. That is, for a perfect cone with maximum *h_undeformed_* = 15 mm (total height of the built DIW cone for our hydrogels), the necessary printed strength to avoid any part yielded or deformed at the bottom should be $\sigma_{\;y}^{p}$ = 147 Pa, considering a material density of 1 g/cm^3^. Figure 2b shows the slump cones printed for CMC-PEG-A8 and CMC-GLY-A8 hydrogels. As expected, both formulations were able to be printed into recognizable slump cones, indicating that material has yield strength after being printed. However, due to buckling, imprecise control of the flow rate, or other experimental problems, the printed slump cones show substantial irregularities. The printing yield stress of these materials cannot be calculated without some ambiguity. In both formulations, the upper part of the cone exhibited distorted geometry, while the bottom remained unaffected. Specifically, the CMC-PEG-A8 formulation demonstrated preservation of the 10-degree angle at the base, accompanied by well-defined layers. This suggests that the sample was either slightly or not at all yielded, and it can be assumed that a preliminary estimation of the printing yield stress could consider that the entire cone height remains non-deformed, with an approximate measurement of 15 mm and a yield strength of $\sigma_{\;y}^{p}$ = 147 Pa (as the observed irregularities are not gravity-induced). The printed cone of CMC-GLY-A8 exhibits additional peculiarities. The cone base is slightly deformed, and the layers are less distinct. This shape results in a smaller undeformed value of 8.2 mm, leading to a lower yield-stress value of $\sigma_{\;y}^{p}$ = 80.5 Pa.

Despite the observed ambiguities, the calculated values of 3D-printable yield strength are relevant parameters to consider as part of our rheological characterization and will be discussed in the context of the subsequent rheological study. Indeed, this innovative in situ approach to post-print flow behavior provides a valuable opportunity to explore the relationship between rheology and printability, a crucial link for improving predictive flow models in order to refine rheological criteria suitable for 3D-printing applications.

### 2.3. Basic Rheological Characterization: The Cox–Merz Rule

As observed, the printability of the hydrogels is determined by their intrinsic composition and the interaction with atenolol. Regarding atenolol distribution, this hydrophilic drug is soluble in the medium (approximately 15 mg/mL), but any excess forms crystalline particles that may be encapsulated within the hydrogel structure, remain in the liquid phase (water or glycerol), or both. CMC-PEG and CMC-GLY formulations containing atenolol appeared stable and homogeneous. Moreover, as previously discussed, the presence of solid atenolol particles at concentrations exceeding the solubility limit played a crucial role in enhancing 3D printability.

From a rheological perspective, our first observation is that the interactions between these biopolymers and atenolol particles at high concentrations (8 wt%) resulted in a thick mixture. However, predicting how this affects both structural changes in the hydrogel network and printability remains a complex challenge. While much research has focused on 3D-printing inks containing spherical micro- or nanoparticles, anisometric particles introduce additional effects. Their interaction with the surrounding medium is significantly different, as they can be oriented and make different contributions to viscosity. In addition, particle shape strongly influences the interactions, with non-spherical particles having a greater tendency to interact due to their higher specific surface area [54,55].

As shown in Section 4, the atenolol particles in the CMC-GLY-A8 hydrogel exhibited a broad size distribution, ranging from 1 to 5 microns, with irregular shapes. These entrapped particles would play a critical role in modulating the rheological properties relevant to printability. However, atenolol particles were not visible in the CMC-PEG-A8 hydrogel by SEM, and we can only speculate that probably it is due to their complete embedding within the hydrogel matrix.

Given the importance of the solid atenolol particles to the printability changes observed in this study, we acknowledge the necessity for their thorough characterization in terms of size and distribution. Our future work will focus on complementary techniques to gain a deeper understanding of the presence and distribution of atenolol within the hydrogel, which is crucial not only for rheology and printability but also for drug delivery performance. Consequently, the rheological characterization presented below, which includes both fundamental and advanced viscoelastic analysis, will be interpreted with caution, emphasizing the most relevant aspects for understanding printability performance.

**Figure 3:**
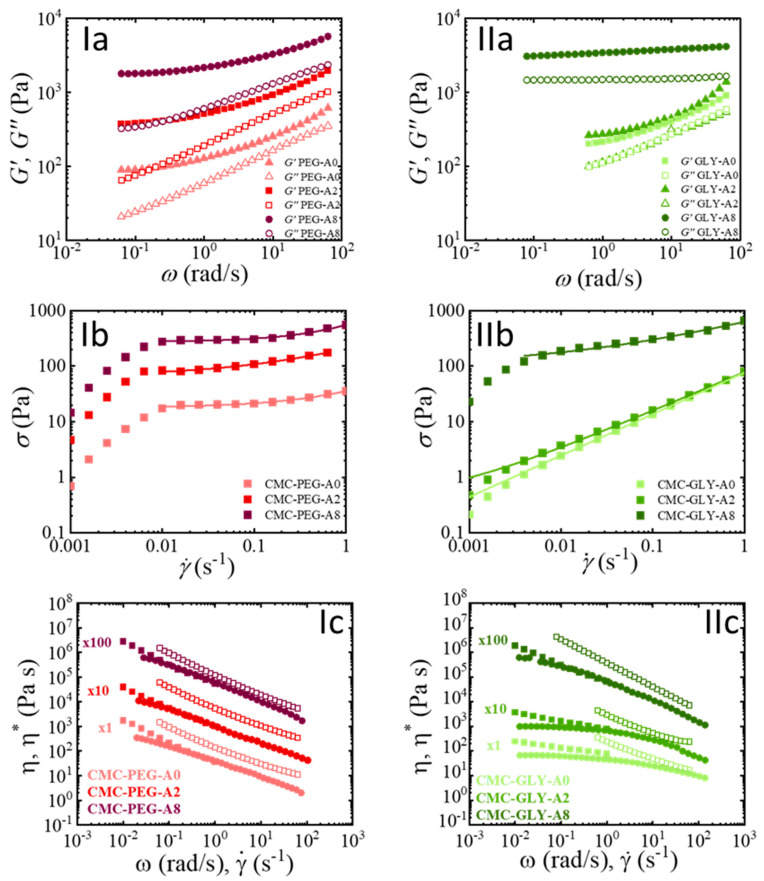
Comparison of the basic rheology of CMC-based formulations containing atenolol A0, A2, and A8. (**Panel I**) CMC-PEG hydrogels and (**Panel II**) CMC-GLY hydrogels. (**a**) The storage modulus, G′, and loss modulus, G″, obtained from the SAOS test in the linear viscoelastic regime. (**b**) Flow curves (shear stress versus shear rate obtained in the range from 0.001 to 1 s^−1^). The experimental data appear as symbols, and the solid lines represent the Herschel–Bulkley model, as indicated in Equation (2) and (**c**). Viscosity from steady-shear test within the range from 0.001 to 1 s^−1^ (filled square symbols) and in the range from 0.01 to 100 s^−1^ (filled circle symbols), and complex viscosity obtained from SAOS (empty square symbols) plotted as a function of shear rate (s^−1^) and frequency (rad/s), respectively. The hydrogel formulations are represented using the same colors in all the figures: PEG-A0, PEG-A2, PEG-A8, GLY-A0, GLY-A2 and GLY-A8.

The basic rheological characterization of hydrogels was performed using two key rheological tests: the oscillatory shear in the linear regime and the steady-shear flow testing. Oscillatory shear in the linear regime (SAOS) data, shown in Figure 3a, analyze the material’s response over a range of frequencies. The storage modulus (*G′*) and loss modulus (*G″*) allow us to gain an understanding of the material’s ability to resist deformation and its overall structural integrity under different frequencies of applied oscillatory shear. The steady-shear flow curve, shown in Figure 3b,c, is a measure of the material’s response over a range of shear rates, simulating conditions similar to those experienced during the extrusion process in 3D printing. Both tests, therefore, characterize the rheological properties of the hydrogel from its behavior at rest, providing information on the strength and stability of the hydrogel ink, together with its response under shear, relevant to its performance during the 3D-printing extrusion step.

Figure 3a shows that the viscoelastic behavior for all CMC-based hydrogels is solid-like, (De > 1), and the storage modulus, G′, is higher than the loss modulus, G″, in the experimental frequency range. CMC-PEG hydrogel formulations show slight dependence on frequency, with moduli G′ and G″ approaching a plateau value for small frequencies. The addition of atenolol increases the moduli values, but the spectrum remains unchanged, as the atenolol interactions seem to not affect the frequency-dependent viscoelastic behavior of the CMC-PEG hydrogel. It can be assumed that atenolol molecules likely reinforce the hydrogel’s elastic structure by interacting with the polymer components, but the primary structural integrity is still governed by CMC-PEG interactions, with atenolol particles homogeneously dispersed within the matrix. The highly structured hydrogel prevents aggregation, maintaining a stable, homogeneous network and improving mechanical properties. The effect of the atenolol content in the CMC-GLY hydrogels follows a different trend. CMC-GLY-A0 and CMC-GLY-A2 show a similar behavior, but CMC-GLY-A8 shows a noticeable increase in moduli, which become frequency-independent, confirming its solid-like behavior reinforced by the dominating effect of the atenolol particles. The different dispersion state of the atenolol particles in the CMC-GLY-A8 hydrogel should be considered. The dispersion of the particles was facilitated by the lubricating effect of glycerol in the hydrogel network, and the enhanced mobility of CMC may contribute to a distinct rheological response under flow conditions, as explained below.

The steady-shear flow curve shown in Figure 3b,c was determined by two procedures within different shear rate ranges. The first shear range, 0.001–1 s^−1^, used for yield-stress determination, and the second range extended to higher shear rates up to 100 s^−1^ to experimentally measure viscosities under typical extrusion conditions. These experiments were carried out by increasing the shear rate values from low to high shear rates under steady-shear conditions. The observed differences in viscosity values in the low shear rate zone following these two procedures could be explained by different equilibrium structures reached for the hydrogels during testing. This undoubtedly highlights the complexity of the characterization of these materials, whose structures evolve slowly over time, making it challenging to determine the equilibrium state.

The stress (*σ*) versus shear rate ($\dot{\gamma }$) data in the lower shear rate range, in Figure 3b, show the transition from solid-like to liquid-like behavior, with two distinct responses identified as the Hookean solid regime (0.001–0.01 s^−1^) preceding the yielding response (0.01 to 1 s^−1^). The data corresponding to the Hookean response were omitted in Figure 3c for clarity. The Herschel–Bulkley model (Equation (2)) is effective for characterizing the material’s response within the shear rate range of the yielding response, allowing for the determination of key rheological parameters, such as the yield stress ($\sigma_{y(H-B)}$), consistency index (k), and flow index (n) [56]. These parameters are summarized in Table 1.

(2) $$\sigma \left(\dot{\gamma }\right)=\sigma_{y(H-B)}+k{\dot{\gamma }}^{n}$$

Although the oscillatory regime indicated a gel response in all CMC-based hydrogels, the flow curve of CMC-GLY-A0 and CMC-GLY-A2 exhibit a shear thinning behavior with no yield stress, suggesting that its structure was notably weaker and more sensitive to shear. Additionally, the lower than expected yield stress observed for CMC-GLY-A8 further indicates that shear forces significantly weaken the intermolecular interactions within the CMC hydrogel, leading to the deterioration of the hydrogel structure. This behavior further emphasizes the distinct rheological characteristics of the two hydrogels’ families.

The steady-shear viscosity (η) and complex-viscosity ($\eta^{*}$) are commonly correlated using the Cox–Merz empirical rule, Equation (3). This widely applied relationship enables the estimation of high shear rate behavior from oscillatory viscosity measurements, as an essential approach when steady-shear experiments become unreliable due to edge fractures or other instabilities.

(3) $${\left[\eta^{*}(\omega )\right]}_{\omega =\dot{\gamma }}=\eta (\dot{\gamma })$$

However, as shown in Figure 3c, the Cox–Merz rule does not hold for these hydrogels. The deviation was anticipated, as such behavior is commonly observed in yield-stress fluids, including highly concentrated suspensions, polymer composites, and hydrogel systems [42]. To address such cases, a modification known as the Rutgers–Delaware rule has been proposed [57]. The approach expresses the complex viscosity as a function of the oscillatory apparent shear rate, $\omega \gamma_{0}$.

(4) $$\eta^{*}\left(\omega \gamma_{0}\right)=\eta (\dot{\gamma })$$

This relationship is expected to hold when the applied deformation is sufficient for the material to yield [57]. Therefore, oscillatory nonlinear experiments performed at strains where the transition from an elastic solid to a liquid state occurs have received considerable interest in relation to analyzing yield-stress behavior. The next section is devoted to the study of the oscillatory nonlinear characterization at high strain so that we can contemplate the equivalence of oscillatory and steady-shear flow under these conditions.

### 2.4. Nonlinear Viscoelastic Characterization Using LAOS

**Figure 4:**
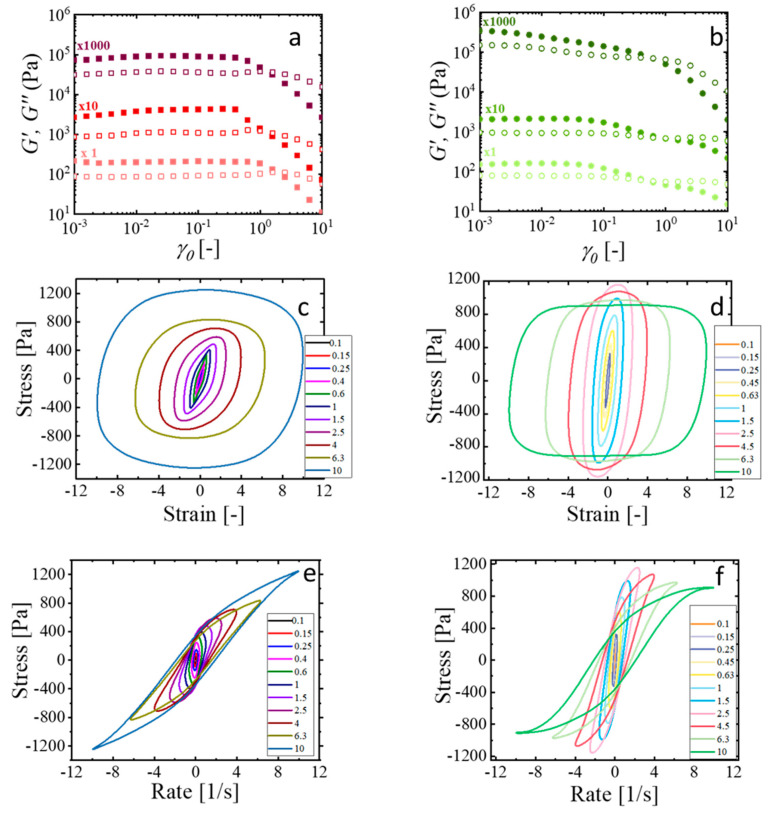
Comparison of LAOS data obtained at frequency of 1 rad/s for CMC-based hydrogels. CMC-PEG data are on the left side, and CMC-GLY data are on the right side. (**a**,**b**) Viscoelastic moduli, *G′* (filled symbols) and *G″* (empty symbols), vs. strain amplitude, $\gamma_{0}$. The hydrogel formulations are represented by the following colors: CMC-PEG-A0, CMC-PEG-A2, CMC-PEG-A8, CMC-GLY-A0, CMC-GLY-A2, CMC-GLY-A8. (**c**,**d**) L-B elastic curves. (**e**,**f**) L-B Viscous curves. Different colors indicate the applied strain amplitude, $\gamma_{0}$.

The use of LAOS is particularly relevant for studying yielding phenomena, as it enables the investigation of nonlinear processes that govern the transition between solid-like and liquid-like behavior under a high strain. The response of the CMC-PEG and CMC-GLY hydrogels to the oscillatory strain sweep test at an angular frequency of 1 rad/s is shown in Figure 4a,b. From the data of Figure 3a,b, it is evident that the viscoelastic behavior at this frequency corresponds to large *De* numbers, because *G′* is larger than *G″* for all the investigated hydrogels. Further, strain amplitudes were applied up to 1000% (10 strain units), which generates shear rates of $\dot{\gamma }=1$0 s^−1^ that fall within the central region of the flow curve, corresponding to the yielded region of viscosity, as observed in Figure 3c,d. The corresponding elastic and viscous L-B representations are shown in Figure 4c–f for the materials CMC-PEG A8 and CMC-GLY-A8, selected as examples of hydrogels with the best printability. Elastic L-B figures are generated by plotting stress against strain (clockwise path), and viscous L-B figures are obtained by plotting stress against the rate (counterclockwise path).

The following analysis of the LAOS data will focus on three key points: (1) The quantification of the intrinsic nonlinear viscoelasticity using FTR. This stablished approach identifies higher-order harmonics in the stress response by transforming the stress signal into the frequency domain. (2) The identification of the nature of the nonlinearities from both the elastic and viscous components of the material’s response allows for a clearer understanding of how each contributes to the material’s overall behavior under strain. This study is performed through the decomposition of the stress signal into its elastic and viscous components, using Chebyshev Polynomials (FTR-SD). (3) Physical interpretation of the yielding process by the SPP framework involves the quantitative analysis of the Lissajous–Bowditch curves to extract information about transient nonlinearities.

#### 2.4.1. Quantification of the Intrinsic Nonlinear Viscoelasticity

As observed in Figure 4a,b, the solid-to-liquid transition of these hydrogels occurs by increasing oscillatory strain amplitudes. In the SAOS regime, the hydrogels exhibit solid-like behavior, with the storage modulus, *G′*, remaining relatively constant and much larger than the loss modulus, *G″*. As the strain amplitude increases, *G′* gradually decreases, eventually crossing *G″*, indicating the transition to a liquid-like state in the LAOS regime. The strain amplitude at which the moduli *G′* and *G″* deviate from their constant, strain- independent value was characterized by FTR analysis.

**Figure 5:**
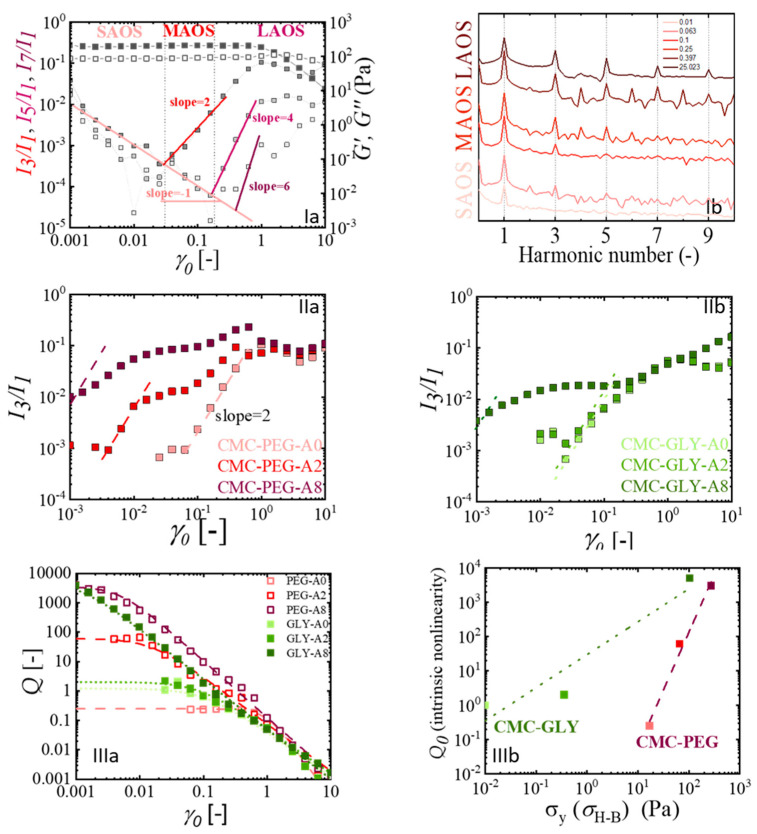
Analysis of the oscillatory strain sweeps test of the hydrogels subjected to a frequency of 1 rad/s from FTR rheology (TRIOS v5.1 software (TA Instruments, New Castle, DE, USA)). (**I**) CMC-PEG-A0 hydrogel data describe viscoelastic SAOS, MAOS, and LAOS regimes**.** (**a**) *G′* and G″, are the moduli calculated from traditional oscillatory equations. The dependence of the nonlinear harmonic parameters, *I*_n/1_, on the strain amplitude is shown. (**b**) Stress signal spectra for different strain amplitudes allow for the detection of the first occurrence of the 3rd, 5th, 7th, and 9th harmonics. (**II**) Nonlinear 3rd harmonic intensity, *I*_3/1_, calculated for atenolol containing hydrogels: (**a**) CMC-PEG hydrogels and (**b**) CMC-GLY hydrogels. (**III**) Calculation of the nonlinear zero-strain, $Q_{0}$, intrinsic nonlinear parameter. (**a**) Nonlinear Q parameter as a function of strain amplitude allow us to calculate the $Q_{0}$ parameter trough a Carreau-like equation: $Q=Q_{0}/(1+b\gamma^{a})$ (the lines correspond to the fitting of the model). (**b**) Nonlinear $Q_{0}$ parameter versus the yield stress obtained from Herschel–Bulkley model of the steady-shear curve, σ_y(H-B)_. Solid lines represent linear fits of $Q_{0}$ versus σ_y(H-B)_.

The linear-to-nonlinear viscoelastic transition marks the point at which the material’s stress response shifts from a sinusoidal pattern to a more complex non-sinusoidal behavior with higher harmonics. As observed in Figure 5Ia for the sample CMC-PEG-A0, the nonlinear regime is indicated by the appearance of the third harmonic in the stress signal. This is followed by additional odd harmonics (e.g., fifth, seventh, etc.) which display similar increase in intensity, but with lower overall magnitudes as strain increases. Figure 5Ib allows us to identify an intermediate strain regime, the medium amplitude oscillatory shear (MAOS), where the third harmonic intensity normalized by the first harmonic (I_3_/I_1_) emerges as the key nonlinear parameter because it is the dominant higher harmonic signal. The MAOS regime simplifies the complexity of the nonlinear rheological response compared to regimes with a more extensive harmonic spectrum and provides more comprehensive insights than the SAOS regime, which is purely linear.

Therefore, we investigated the MAOS region to delve deeper into the influence of the hydrogel’s microstructure on its rheological properties. The analysis was found particularly useful for detecting the boundary between linear and nonlinear response, where the third harmonic is the sole higher harmonic present in the stress signal. Therefore, our first approach to the analysis of nonlinear oscillatory data will focus on the *I*_3_/*I*_1_ magnitude. This factor can be considered the most significant in controlling waveforms, as successive higher-order harmonics tend to decay more rapidly. In addition, the transition from the linear to nonlinear region is captured by this parameter because it is highly sensitive to small changes in the modulus values; even a 10% change in moduli can result in a significant increase in the third harmonic intensity—by several tens of times, as cited in Reference [58]. The decrease in the moduli and the increase in the intensity of the third harmonic occur at different strain amplitudes for the CMC-based hydrogels, highlighting the structural differences due to the hydrogel formulations and the atenolol content, as can be observed in Figure 5II.

We analyzed the nonlinear response in the MAOS region using the concept of intrinsic nonlinearity. The methodology, proposed by Hyun et al. [59], is based on the experimental observation that the third harmonic intensity, I_3/1_ (ω,γ_0_) = σ_3_/σ_1_, i.e., the intensity of the third harmonic, σ_3_, normalized by the intensity of the fundamental, σ_1_, scales quadratically with the strain amplitude, γ_0_, in the MAOS regime, I_3/1_~γ_0_^2^. The relationship for the relative intensity, I_n/1_, can be expanded in powers of the shear strain amplitude, so that In1~γ0n−1 with n = 1, 3, 5, … as it is nicely observed for hydrogel CMC-PEG-A0 in Figure 5Ia. However, a detailed analysis of the odd higher harmonics (the fifth, seventh, etc.) in the LAOS region is beyond the scope of the present work; therefore, the focus will be exclusively on the third harmonic signal. Figure 5IIa-b show the I_3/1_ ~ γ_0_ results obtained for all the CMC hydrogels.

We calculate the nonlinear parameter, Q, defined as $Q=\frac{I_{3/1}}{{\gamma_{0}}^{2}}$. This parameter when evaluated at relatively small strains, results in a constant value (similar to Newtonian viscosity), which allows us to define a zero-strain nonlinear parameter, $Q_{0}$, as $\underset{\gamma_{0}\to 0}{\lim}Q\equiv Q_{0}$. The constant value of $Q_{0}$ does not mean that nonlinearity disappears but that it remains constant at low strains, so the $Q_{0}$ coefficient reflects inherent nonlinear properties of the materials. Therefore, $Q_{0}$ is a parameter obtained from the intensity of the third harmonic in MAOS. In this region, as discussed earlier, the third harmonic is the main signal with sufficient intensity to allow a physical interpretation of the behavior, and it is also a region where the experimental errors generally observed for larger deformations (wall slip, secondary flow, and inertial effects) are minimized. The nonlinear parameters, $Q\left(\gamma_{0,}\omega \right)$, for all CMC hydrogels are shown in Figure 5IIIa. In this plot, the strain amplitude is expressed as absolute strain amplitude. The obtained numerical values for $Q_{0}$ were determined using a Carreau-like equation, $Q=Q_{0}\left(1+b\gamma^{a}\right)$. CMC-PEG-A8 and CMC-GLY–A8 hydrogels exhibit the highest value of $Q_{0}$ (hence being more nonlinear), followed by CMC-PEG-A2 and CMC-GLY-A2, and then CMCGLY-A0 and CMC-PEG-A0. It was reasonable to hypothesize that these variations would be related to the differences in non-Newtonian behavior and yield stress, which were more pronounced as the weight fraction of atenolol particles increased.

It is important to note that the nonlinear parameter $Q_{0}$ is considered a critical indicator for assessing the structural behavior of complex materials. Its sensitivity to phase distribution, particle size, and other factors related to microstructure and architecture has been extensively described in polymeric systems. Thus, $Q_{0}$ has been used in several systems to understand how different components influence the internal structure and properties of a material. For example, in nanocomposites, including those formulated with hydrogels, the incorporation of carbon nanotubes (CNTs) and graphene (Gr) has been reported to increase the I_3_/I_1_ ratio. This suggests that the presence of nanoparticles significantly alters the internal structure, probably affecting the phase distribution and interactions within the material [12]. Similarly, in polymer blends, $Q_{0}$ has been found to be useful in assessing the effect of droplet size on dispersion quality [60]. The application of this analysis extends to food science. In the case of commercial chocolates, the presence of solid particles and their bimodal size distribution, which is closely related to chocolate texture, yield strength, and overall microstructure, has also been found to be significantly related to the $Q_{0}$ parameter [61]. It is therefore expected that the ability to capture the influence of phase interactions and size distribution can be also useful in the evaluation of hydrogels formulated for pharmaceutical applications, such as those included in this work. The viscoelastic intrinsic nonlinearity observed for the CMC-based hydrogels appears to be clearly linked to the presence of atenolol solid particles. The presence of atenolol, which, for 8 wt% of the concentration, is not soluble and forms solid particles of irregular shape (as observed in Section 4.1), increases the strength of the hydrogels. This effect is related to the increase of the intrinsic nonlinear parameter. Figure 5IIIb shows a linear relationship between the nonlinear $Q_{0}$ parameter and the yield stress obtained from Herschel–Bulkley modellization of the steady-shear curve. This trend, obtained for both families of hydrogels investigated, indicates that the $Q_{0}$ and the yield-stress value are affected in the same way by the increase in the content of dispersed solids in these hydrogels, indicative of the strengthening effect within the hydrogel network of the presence of atenolol.

Furthermore, in the LAOS region, the third harmonic FT spectra shown in Figure 5IIa,b, reveal key insights into structural changes within the hydrogel during deformation. For all formulations containing atenolol (not for CMC-GLY-A2), the third harmonic intensity reaches a plateau after the medium strain region that accounts for the effect of the shear on the interactions of hydrogel–atenolol solid particles. In addition, at even higher strains, most samples (CMC-PEGA0, CMC-PEGA2, CMC-PEGA8, CMC-GLYA0, and CMC-GLYA2) display a subsequent maximum which decreases with strain before increasing slightly with further increase in strain amplitude. In contrast, CMC-GLYA8 formulation shows a continuous increase in intensity without the intermediate drop. The “hump” in the third harmonic intensity can be attributed to the shear-induced structuralchanges, as the hydrogel aggregates, oriented and aligned in the flow direction, undergo further evolution. This behavior is similar to that reported for various two-phase systems, including carbon black-filled rubber, concentrated PMMA suspensions in PDMS, rigid polymer dispersions and soft gels [25,62,63,64,65]. Consequently, the increase in intensity after the minimum in our studied hydrogels suggests a microstructural reorganization under flow. Specifically, when strain amplitudes exceed $\gamma_{0}$ > 4, the increase in the third harmonic indicates that the material is capable of reorganizing large aggregates, such that the reformed interactions within the hydrogel structure provide additional flow resistance. The LAOS region, renowned for being able to detect microstructural reorganization in the yielding regime [25], highlights the greater potential for reorganization of the CMC-PEG-A8 hydrogel compared to CMC-GLY-A8 hydrogel.

#### 2.4.2. The Elastic and Viscous Nature of the Nonlinearity

**Figure 6:**
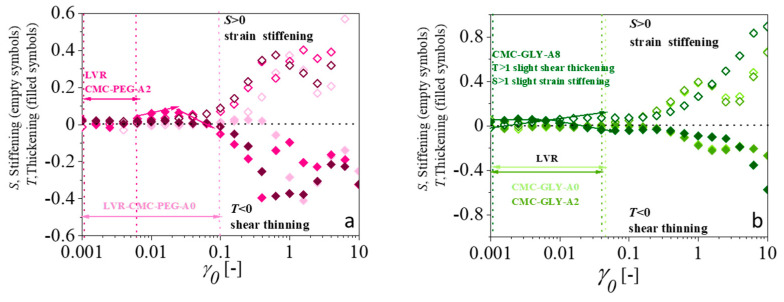
Dimensionless nonlinear coefficients: stiffening (S) and thickening (T) are represented for hydrogels formulations CMC-PEG (**a**) and CMC-GLY (**b**). Vertical dashed lines correspond to the strain for the onset of nonlinearities. S data are empty symbols and T data are filled symbols.CMC-GLY-A0 (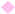, 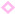), A2 (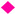, 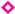) A8 (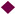, 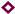); CMC-GLY-A0 (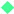, 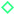)A2 (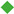, 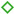), A8 (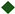, 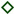).

After the analysis of the intrinsic nonlinear parameter $Q_{0}$ usig FTR analysis, this section focuses on the quantification of the nonlinearity in terms of its viscous and elastic contributions. This is a further step toward understanding the complex rheological response of these hydrogels, marked by the yield-stress transition that occurs before flowing. The methodology, based on the stress decomposition of Lissajous–Bowditch curves, is supported by symmetry arguments, providing a robust mathematical tool to decouple the elastic and viscous components of the periodic stress signal [36,37]. However, the methodology has important limitations because the symmetry can be lost, especially for materials that change with time, start-up flows, or transient phenomena. In fact, the SD method, when applied to complex systems like hydrogels, requires careful interpretations [61]. Experimentally, all LAOS data of the CMC hydrogels were carefully revised to discard transient behaviors, ensuring that all the assumptions of the method were met. The closed stress–strain and stress-strain rate loops, generated at different fixed strain amplitudes, were analyzed to provide viscous and elastic nonlinearities. The application of the method enabled us the determination of the well-known shear-thinning–shear-thickening and strain-softening–strain-stiffening responses during the viscoelastic transition that otherwise would require performing separate experiments [36,37]. Figure 6 shows, for a wide range of selected strains, the stiffening, S, and thickening, T, parameters, calculated as described in the Materials and Method section. These values are plotted for CMC-PEG (Figure 6a) and CMC-GLY (Figure 6b) hydrogels. The horizontal lines in these figures represent linear rheological behavior (S = 0 or T = 0) and nonlinear behavior above or below it.

CMC-based A0 hydrogels exhibit a largely linear viscoelastic LVR region, as observed in the nonlinear parameters S and T vs. *γ*_0_ plots, with S = 0 and T = 0. As strain increases, a pronounced transition to the nonlinear regime occurs. The S parameter becomes positive, indicating elastic nonlinear stiffening behavior, while the T parameter becomes negative, reflecting viscous nonlinear shear thinning. The steep slope indicates significant changes in the hydrogel network in this region, corresponding to the medium-amplitude oscillatory shear, MAOS, region. At higher strain levels, close to the region of the characteristic intersection, *G′ = G″*, the nonlinearity profile undergoes further transitions. Specifically, the S parameter reaches a maximum, and the T parameter a minimum, indicating a decrease in the elastic nonlinearity intensity and a plateau in the viscous contribution, consistent with the fluid-like behavior of this region. As discussed in the previous section, closely related to the observed behavior reported for the third harmonic signal of the stress, following the S-peak–T-valley transition region, the further increase in strain caused the stiffening, S, parameter to increase and the thickening, T, parameter to decrease. The overall trend remains consistent across the different CMC-based formulations. For the CMC-based A2 formulations, the response is qualitatively similar but with some specific differences. The CMC-PEG-A2 formulation shows a slight nonlinear viscous thickening at very low strains, possibly related to weak interactions between the hydrogel components.

The CMC-GLY-A8 and CMC-PEG-A8 formulations display distinctive behaviors. Both lack a clear linear viscoelastic region, even at low amplitudes, suggesting a significant role of the atenolol solid particles on nonlinear responses. For CMC-PEG-A8, the behavior is similar to that of the CMC-PEG-A0 and CMC-PEG-A2 hydrogels, but nonlinearities in the MAOS region—strain stiffening and shear thinning—emerge at lower strain levels. In the LAOS region, overlapping of the S and T parameters is observed. In contrast, the CMC-GLY-A8 formulation differs markedly, within the MAOS region it is observed slight strain stiffening and viscous thickening, which transitions to shear thinning as strain increases. Near the crossing point, *G′ = G″*, the maxima in S and minima in T are absent. Instead, both elastic and viscous nonlinearities increase noticeably.

These quantitative differences suggest that not all the yielding processes are the same among the formulations investigated. The incorporation of atenolol solid particles in the A8 formulations produces different impact on the elastic and viscous components of the stress. The addition of atenolol to the CMC-PEG hydrogels results in nonlinear coefficients evolving during flow similarly to those of particle-free formulations, implying that viscoelasticity during yielding is primarily governed by interactions between CMC and PEG components. Structural reorganization during yielding leads to the observed thinning–thickening–thinning/stiffening–softening–stiffening transitions. These findings indicate that the incorporation of atenolol does not appear to hinder this behavior. Conversely, atenolol addition to CMC-GLY hydrogel enhances both elastic strain stiffening and viscous shear thinning, indicating a stronger influence of the atenolol particles’ within the hydrogel network, as viscoelastic transitions are not observed at high strain levels. In other words, the presence of solid atenolol particles inhibited the structure reorganization of the CMC-GLY hydrogel during flow, whereas the CMC-PEG hydrogel preserved its ability to reorganize.

#### 2.4.3. Physical Approach to the Yielding Process

The SPP framework was quantitatively applied to investigate the time-resolved nonlinear viscoelastic properties of the CMC-based hydrogels. The methodology is summarized in the Materials and Methods section, with the main concept being that the stress response at any given point in time is viewed as a combination of different physical processes occurring sequentially [38,39]. Mathematically, the stress signal at a specific moment is represented as the sum of contributions from both elastic (instantaneous) and viscoelastic (time-dependent) responses, as expressed by Equations (5)–(7).

(5) $$\sigma \left(t\right)=G_{t}^{\prime }\gamma \left(t\right)+G_{t}^{\prime \prime }\left(t\right)\;\dot{\gamma }\left(t\right)/\omega +\sigma^{d}(t)$$

(6) $$G_{t}^{\prime }\left(t\right)=\frac{\partial \sigma \left(t\right)}{\partial \gamma \left(t\right)}$$

(7) $$G_{t}^{\prime \prime }\left(t\right)=\omega \frac{\partial \sigma \left(t\right)}{\partial \dot{\gamma }\left(t\right)}$$

where $G_{t}^{\prime }\left(t\right)$ and $G_{t}^{\prime \prime }\left(t\right)$ represent the instantaneous dynamic moduli defined using the partial derivatives of the transient response, and *σ^d^* is the displacement stress, which physically accounts for yield stresses [22,38].

The advantage of the SPP is that it does not rely on selecting discrete values of specific strains and strain rates. Instead, all strain, strain rate, and stress components along the L-B curve are evaluated. A significant body of work has been developed within the SPP framework to explain various flow behaviors, with particular significance arising from the rigorous analysis devoted to the yielding phenomena. Integrating this understanding into the analysis of 3D-printable hydrogels becomes particularly relevant when discussing the rheological criteria that govern their printability [11,12,18,22]. In fact, the SPP analysis facilitates the examination of the rheological transitions within the oscillation cycle using the transient Cole–Cole $(G_{t}^{\prime \prime }\left(t\right)$ vs. $G_{t}^{\prime }\left(t\right)$) plots [23,37,39,40,41,66,67,68]. Changes in the elasticity are specifically accounted for by $G_{t}^{\prime }\left(t\right)$, indicating stiffening or softening behaviour, while the viscous changes are accounted for by $G_{t\;}^{\prime \prime }\left(t\right)$, indicating thickening or thinning. The solid-like viscoelastic behavior is defined at times where $G_{t}^{\prime }\left(t\right)>G_{t\;}^{\prime \prime }\left(t\right),$ while the reverse indicates liquid-like viscoelasticity. Fluidizing or solidifying transitions occur across the $G_{t}^{\prime }\left(t\right)=G_{t\;}^{\prime \prime }\left(t\right)$ line. Consequently, the intra-cycle response will inform about the microstructure evolution of the hydrogel during the solid-to-liquid transitions occurring at a specific strain amplitude, and the variations in area and location of trajectories at increasing strain amplitudes provide information on inter-cycle rheological transitions.

**Figure 7:**
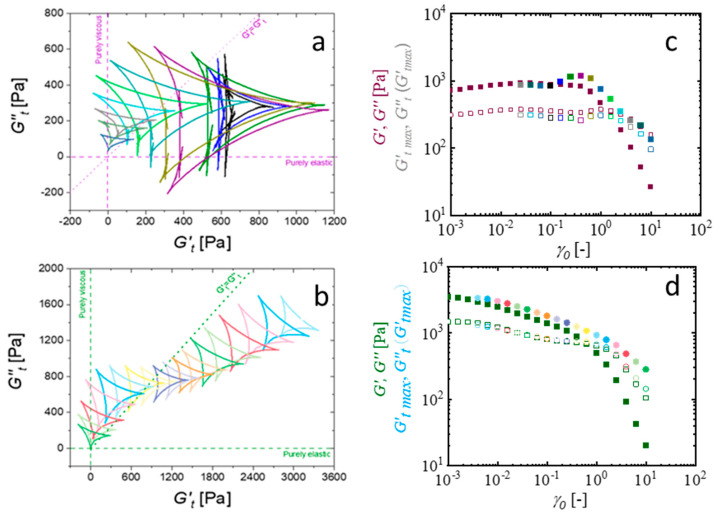
SPP analysis of the rheological transition at various strain amplitudes using Cole–Cole plot for CMC-based hydrogels containing atenolol A8. (**a**,**b**) The trajectories of the transient Cole–Cole plots take the form of deltoid-type curves plotted counterclockwise. The dashed lines represent *G′_t_* = *G″_t_*, purely elastic response at *G′_t_* = 0, and purely viscous response at *G″_t_* = 0. (**a**) CMC-PEG-A8 and (**b**) CMC-GLY-A8. (**c**,**d**) Fundamental moduli (G′, G″) and SPP moduli at the point of maximum elasticity (*G′_tmax_*, *G″_t_* (*G′_tmax_*). (**c**) CMC-PEG-A8 and (**d**) CMC-GLY-A8. Storage modulus, *G′* are represented with filled symbols and loss modulus, *G″*, with empty symbols. Cole-Cole plot and the G′_tmax_ and G″_t_ (G′_tmax_) LAOS data are identified with the same colors for the different strain amplitudes, $\gamma_{0}$.

Within the region of linear viscoelasticity, structures remain in equilibrium, resulting in Cole–Cole plots with a small, reduced area. Conversely, as deformation initiates and the material transitions into nonlinear behavior, extensive structural reorganization occurs. This reorganization manifests as larger deltoids in the Cole-Cole plots, reflecting increased energy dissipation. Interestingly, as observed in Figure 7a,b, the evolution of the deltoid’s size and orientation with increasing strain amplitude for the formulations with better printability, CMC-PEG-A8 and CMC-GLYA8, shows different trends that quantify different yielding transitions. For the CMC-PEG structured hydrogel, the area enclosed by the deltoids remains very small at the lowest strain amplitudes (data obtained in the range $\gamma_{0}$ = 0.001–0.01 are not included due to noise and some distortions), but increases considerably from $\gamma_{0}=0.1$ up to about $\gamma_{0}=1$. This increase in area indicates strain-induced changes in the microstructure as the hydrogels leave the LVR. In fact, the deltoids progressively increase in size by encapsulating the smaller LVR deltoids, until a maximum size is reached before entering the flow region (corresponding to a *G″ > G′* behavior). In this region, the structural rearrangements become less pronounced (smaller deltoids) due to the microstructural degradation, or network damage, as the strain amplitude increases further toward the LAOS, leading to a weakening of the nonlinear response. In contrast, for the CMC-GLY structured hydrogel the LVR region is not clearly observed, and the deltoid area remains almost unchanged over the broad region of the applied strain, with only a slight increase observed in the region near $\gamma_{0}=0.01$ and in the region of crossover point (*G′ = G″*), near ${\;\gamma }_{0}=1$. As the strain amplitude increases further, the deltoid areas eventually evolve similarly to that observed in CMC-PEG hydrogels, with a weakened nonlinear response, leading to deltoids with even smaller areas. These Cole–Cole plots demonstrate the different structural evolutions during the solid–liquid transition (“flow”) experienced by formulations with high concentrations of atenolol A8.

The evolution of the deltoid size with increasing strain amplitudes demonstrates the formation of reinforced elastic structures in the CMC-PEG-A8 hydrogel before flow, in contrast to the degradation of the CMC-GLY-A8 microstructures observed almost from the beginning of the amplitude sweep. The presence of atenolol particles, identified as the critical factor to ensure the necessary strength for printability, has a different effect on the deformation of the hydrogel structure of the CMC-PEG and CMC-GLY systems. Therefore, analysis of the SPP Cole–Cole plot in terms of physical interpretation is expected to provide complementary information to elucidate these differences, as addressed below.

The following discussion aims to use the capabilities of SPP analysis to understand how hydrogel structures adapted to applied strain. The objective is to evaluate the hydrogels’ intra-cycle structural breakdown and flow, as well as their recovery capacity once the deformation rate is reduced to zero. These behaviors are crucial for predicting the key properties necessary for optimizing printability. To this end, a thorough analysis of the transient evolution of moduli under deformation will be conducted, with a focus on two critical parameters: the cage modulus and the elastic deformation.

The elastic nature of the hydrogel structure at rest is quantified by the storage modulus, *G′*, measured within the linear viscoelastic region (see Section 2.1). To extend this concept into the nonlinear viscoelastic conditions, the definition of the cage modulus, *G_cage_* was introduced [69]. The original cage modulus, *G_cage_*, was derived from the notion that the stress–strain relationship at the zero-stress point in the elastic L-B curves expressed the linear viscoelastic modulus of the cages, where the cages (or structural units) undergo linear-elastic extension. This metric has been employed in numerous systems, thereby corroborating the correlation between the cage modulus and the linear viscoelastic modulus, *G′* [11,67,68,69,70].

Taking advantage of the quantitative approach of SPP, the elastic response of the reformed cages at each oscillation cycle was quantified directly using the SPP Cole–Cole plots from the point of maximum elasticity located where *G′_t_* = *G′_tmax_* in cases where G′_t_ >> *G″t* [38]. In fact, *G′_tmax_* is considered an approximation to the cage modulus, *G_cage_*, since the point of maximum elasticity represents not the purely elastic behavior of the structure, but its viscoelastic nature. This is observed in Figure 7c,d, where the viscous modulus, *G″_t_*, at the point of maximum elasticity, G′_tmax_, is non-zero but very close to the value of the fundamental G″. Structural cages do not extend in a purely elastic manner; rather, they extend viscoelastically. The clear correspondence between the maximum elasticity and the linear viscoelastic regime suggests that the elastic physical processes occurring in the nonlinear and linear regime are similar in our systems. However, the observed decrease in the modulus of elasticity, *G′_tmax_*, as the material flows (G″ > G′) informs us about the structures’ memory being lost.

Interestingly, the SPP framework reveals that within a single deformation cycle, even under high-strain-amplitude conditions, the materials transition from a predominantly elastic response at low strain, *G′_t_* > *G″_t_*, to a predominantly viscous response at higher strain, *G′_t_* < *G″_t_*. This indicates that the material spends time in elastic and plastic deformation, between yielding and non-yielding processes. This general behavior observed at the higher strain amplitudes allows us to quantify the elastic response even under flow conditions, where the averaged loss modulus G″ dominates. Specifically, the CMC-PEG hydrogel shows both elastic and viscous overshoots, detected with rising strain amplitude by the increase in *G′_tmax_*, and in the corresponding G″_t_ (measured at G′_tmax_). This suggests a structural hardening effect under a certain range of strain ($\gamma_{0}\sim 0.1-1)$. The CMC-GLY shows continuous structural degradation under strain, with a weak plateau of elasticity observed at some intermediate strain, $\gamma_{0}\sim 1.$ These results highlight the complex effect of shear on the atenolol-hydrogel networks. In the CMC-PEG formulation, interactions among CMC, PEG and atenolol enhance the structural strength, creating a robust structure that remains unstable, because it can be recovered, even as the strain amplitude increases. In this hydrogel, *G′* and *G′_tmax_* are strain-independent moduli up to $\gamma_{0}\sim 0.1.$ Near the flow region, where *G′* = *G″*, a slight strain hardening (increase in *G′_tmax_*) and thickening (increase in *G_t_″*) is observed. The behavior was similar to that observed for CMC-PEG-A0 and CMC-PEG-A2, suggesting that although the presence of atenolol increases the modulus of the hydrogel formulation, the viscoelastic transition through the flow region is governed by the hydrogel dynamics. The more complex response of the CMC-GLY hydrogel can be attributed to different kinds of interactions. While the hydrogel structure begins to progressively degrade even at the lower strain level, affecting the yielding mechanism observed in the low strain range, some strong interactions persist at higher strains, leading to partial recovery of the structural strength. Consequently, the modulus at the maximum elastic point, *G′_tmax_*, also remains higher than *G″_t_*, even at the highest applied strain amplitude.

The presence of atenolol in the CMC-PEG hydrogel improves the elasticity, maintaining a good stability of the cage structure. *G′_tmax_* remains stable over a wide range of deformation before the cages start to stiffen, and eventually become damaged, resulting in incomplete recovery after the inter-cycle yielding process. In contrast, while the presence of atenolol in the CMC-GLY hydrogel also increases the elasticity of the hydrogel at the low strain, the continuous drop in the G′ modulus and in the “apparent” *G_cage_* is evident for all the investigated strains. This behavior is similar to that reported for 3D-printable pluronic hydrogels containing graphene, F127-Gr [11]. The cage elasticity of these F127-Gr hydrogels decreased more dramatically for the higher content of graphene, suggesting, according to the authors, that the presence of graphene increased the unrecoverable processes taking place during the deformation of the cages. It can be hypothesized that the yield of CMC-GLY-A8 hydrogels is also subjected to a greater number of unrecoverable processes in comparison to those supported by CMC-PEG-A8 hydrogels.

**Figure 8:**
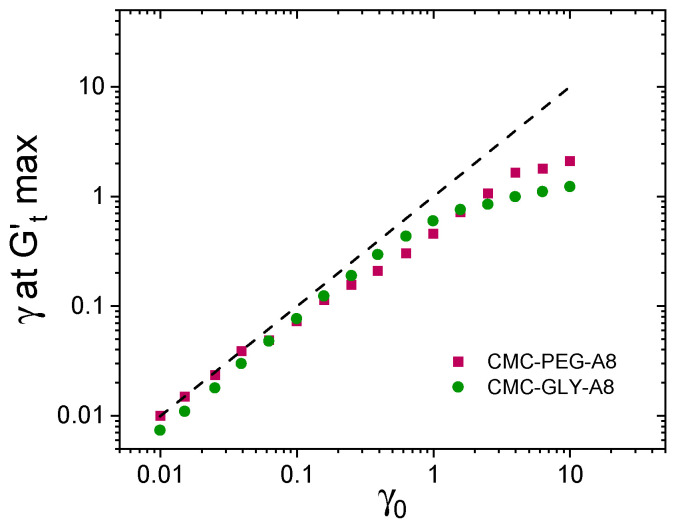
The elastic strain at the point of maximum elasticity determined in SPP Cole–Cole plots, *G′_tmax_*, calculated using quantitative SPP analysis. Dashed lines indicate a perfectly elastic strain equal to the applied strain amplitude. The calculated strains at the point of maximum elasticity are close to the total strain amplitude, $\gamma_{0}$, in the range of $\gamma_{0}\;\sim \;0.01-0.5$.

Additionally, we calculated the strain acquired by the cages in the solid-like viscoelastic region at the point of maximum elasticity, *G′_tmax_*, where $G_{t\;}^{\prime }\left(t\right)>G_{t}^{\prime \prime }\left(t\right)$). Although this calculated elastic strain, $\gamma \;at\;G_{tmax}^{\prime }$, is an indirect measure of the recoverable strain, recent validation has confirmed its strong correlation with data obtained from multiple experimental and analytical approaches, collectively termed Recovery Rheology [42,66]. Figure 8 shows the recoverable-elastic strain, $\gamma \;at\;G_{tmax}^{\prime }$, calculated at the different strain amplitudes. In a purely elastic material, where all the strain is acquired elastically (recoverably), the elastic strain is expected to increase with a slope of 1, indicating perfect memory. This behavior is observed in the CMC-PEG-A8 hydrogel in the range $\gamma_{0}$ = 0.01–0.04. In contrast, CMC-GLY-A8 hydrogel, even at the lowest applied strain is deformed quasi-elastically, as cage deformation processes result in a small but measurable amount of non-recoverable strain. As the strain increases, the cage in both hydrogels experiences more viscous deformation, leading to an increase in the non-recoverable component of the acquired strain. Notably, as demonstrated in Figure 8, at the highest applied strains, the recoverable strain in CMC-PEG hydrogels is observed to be twice that of CMC-GLY-A8 hydrogels.

The apparent cage modulus, *G_cage_*, and the elastic extension of the reformed cages quantified at the position of maximum elasticity, *G^′^_tmax_*, are relevant parameters in the study of the nonlinear response in materials exhibiting yield-stress behavior. These parameters account for the elastic response of the reformed structure at each cycle (or the average energy stored elastically normalized by the recoverable strain amplitude [11,68,69,70]. The cage modulus in Figure 7c,d and the recovered strain in Figure 8 show different trends depending on the structure of the hydrogel based on CMC-GLY or CMC-PEG formulation. The acquired recovered strain in the high strain regime is twice for CMC-PEG-A8 hydrogels than for CMC-GLY-A8 hydrogels, thus affecting the post-yielding behavior of these materials. Enhanced acquisition of recoverable deformation may lead to better recovery of the hydrogel structure after 3D extrusion processes.

**Figure 9:**
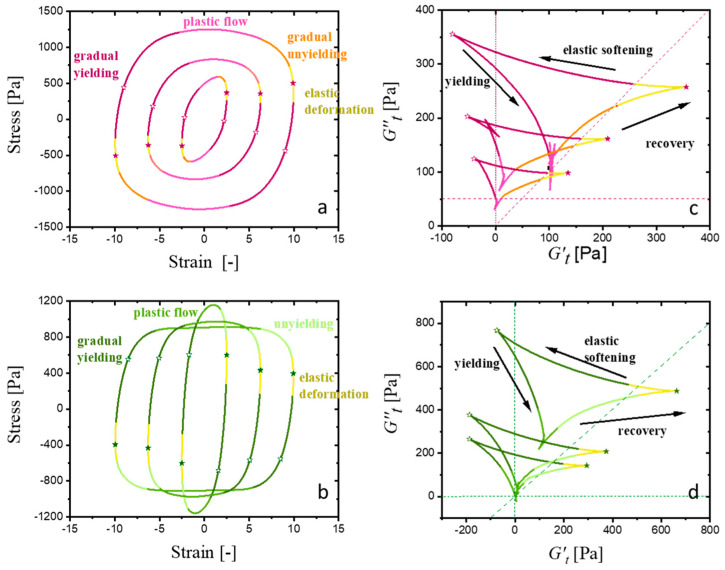
Comparison of SPP data at large strain amplitudes of $\gamma_{0}=2.5,\;6.28,\;and\;10\;$ strain units for CMC-PEG-A8 (upper part) and CMC-GLY-A8 (bottom part). (**a**,**b**) Stress vs. strain, elastic L-B curves, and (**c**,**d**) SPP Cole–Cole plots. Four physical processes are identified from the transitions in the stress–strain curves: elastic deformation, gradual yielding, plastic flow, and gradual unyielding. The critical *G′_tmax_* and *G′_tmin_* parameters are marked with star symbols (*G′_tmax_*, solid-colored star; and *G′_tmin_*, star outline).

Therefore from the point of maximum elasticity, we have obtained the “apparent” modulus of the cages, which is the modulus of the pre-yielding structure, as well as the elastic extension of the cages, by calculating the acquired recoverable strain. Now, it is time to revise the entire sequence of processes to understand how the nonlinear oscillatory data account for the material flows once the cages have been broken. We present in Figure 9 the detailed analysis of the elastic Lissajous and the transient Cole–Cole plots obtained at three selected strain amplitudes, where the yielding–unyielding process are observed. For both hydrogels, CMC-PEG-A8 and CMC-GLY-A8, the stress–strain and *G′_t_* vs. *G″_t_* evolution within a period of oscillation allow us to identify the four physical processes of a yielding transition, as recently proposed by Kamani et al. [22] as (1) elastic deformation; (2) gradual yielding, with intermolecular interactions; (3) plastic flow; and (4) gradual unyielding.

Therefore, in the Cole–Cole plots, these transitions are observed along three trajectories. The main transition is the yielding, characterized by a shift from recoverable processes, where elastic deformation is observed at G′_t_ > G″_t_, to unrecoverable-viscous dominated processes at G′_t_ < G″_t_, eventually leading to plastic flow, where both moduli approach zero (${G\prime }_{t}\sim 0\;and\;{G^{\prime \prime }}_{t}\sim 0)$. During the “recovery” trajectory, the hydrogel structure is recovered progressively, increasing its instantaneous elasticity until it reaches its maximum, *G′_tmax_*, near zero-strain rate and maximum strain. This parameter, *G′_tmax_*_,_ previously described as equivalent to *G_cage_*, represents the modulus of the recovered structure. After the structure recovers, the flow is reversed, and the hydrogels experience strain increasing in the opposite direction. In this trajectory, where *G′_t_ < G″_t_*, it is observed an “elastic softening”, as *G′_t_* decreases until its minimum, *G′_tmin_*, and becomes negative. The decrease in instantaneous elasticity occurs simultaneously with an increase in *G″_t_*, due to the viscous contribution. Next, the structures are deformed with increasing strain rate during the yielding process, where *G′_t_* maintains a negative sign, which can be interpreted as strain-induced structural breakdown due to an excessive increase in strain beyond a critical point [11,44,68]. Finally, the plastic flow condition is observed during a short interval.

**Figure 10:**
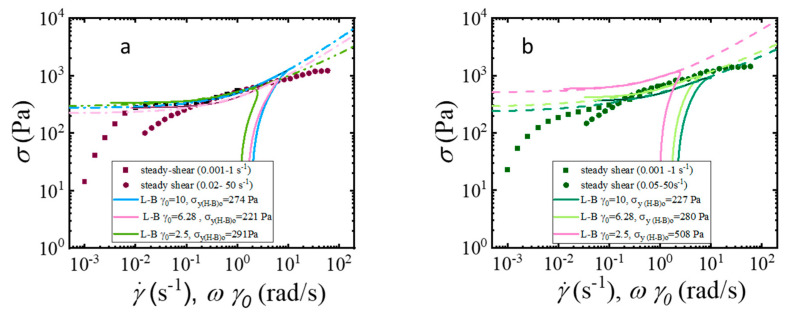
(**a**,**b**) Positive oscillatory stress corresponding to L-B curves for three selected strain amplitudes, $\gamma_{0}=2.5,\;6.28,\;and\;10\;$ strain units (with lines same as in Figure 7) superimposed on the steady-shear flow curve (symbols, same as in Figure 3c). Yield-stress values were calculated using Herschel–Bulkley model fitted to nonlinear positive viscous L-B curves, stress vs. ωγ_0_. (**a**) CMC-PEG-A8, and (**b**) CMC-GLY-A8.

Figure 10 shows the comparison of the stress vs. strain rate relationship obtained from the viscous Lissajous-Bowditch curves and the steady shear flow curves, according to equation 4. The positive oscillatory stress for strain amplitudes $\gamma_{0}=2.5,\;6.28,\;\mathrm{and}\;10$ strain units (with the same line colors than in Figure 7) superimposed quite well onto the steady-shear flow curves (with the same colors as in Figure 3c), for CMC-PEG-A8 and CMC-GLY-A8. This result demonstrates that the response to LAOS covers the full spectrum of rheological behavior, from linear to nonlinear viscoelasticity, with a response comparable to continuous shear flow [71]. The oscillatory yield stress, *σ*_*y*(H-B)o_, is then calculated accordingly. The specific oscillatory data, starting at the higher shear rates, from plastic flow, unyielding, and recovery of elastic strain processes, are nearly identical to those of the steady-flow curve, starting from solid that transition to fluid behavior at stress higher than the yield stress (static yield stress). On the other hand, the value of the modulus at the maximum elasticity, *G′_tmax_*, is the modulus of the recovered elastic structure after flow, corresponding to the dynamic yield stress, the yield stress once the flow stops, with the same physical meaning as the printing yield strength, calculated post-yielding using the DIW slump cone. The oscillatory yield-stress value obtained for CMC-PEG-A8 is similar for the three strain amplitudes investigated, and it is very close to the static yield stress determined from steady-shear measurements (*σ*_*y*(H-B)_ = 275 Pa). However, the oscillatory yield stress calculated for the CMC-GLY-A8 hydrogel decreases as the amplitude of the deformation increases, with noticeable differences compared to the steady-shear experiments (*σ*_*y*(H-B)_ = 104 Pa).

The comparison of the yield-stress parameters determined by the three techniques, steady-shear flow, oscillatory test, and DIW cone slump test, is presented in Table 2.

By measuring the yield stress across these different approaches, the material behavior in each scenario can determine the expectations of the material consistency for printing. Although the results have some differences, the general trends are consistent for the three experimental analyses. In practical terms, comparing the yield stress from these different rheological tests allows us to understand how the hydrogel behaves both under constant shear (steady shear) and under large dynamic shear (LAOS shear), close to the conditions it would experience during 3D printing.

## 3. Conclusions

The results of this work provide a framework for the evaluation of rheological criteria associated with the “printability” of soft materials under DIW. The printability of CMC-based atenolol hydrogels was strongly influenced by the presence of atenolol particles. Although all formulations were “printable”, tablets printed with formulations containing 8 wt% atenolol showed better shape retention than those containing only 2 wt% or no atenolol. This ability to maintain printing resolution during filament deposition is rheologically dependent on the thixotropic character of the samples, as “high recoverable yield stress” was essential for maintaining structural integrity after extrusion.

**Figure 11:**
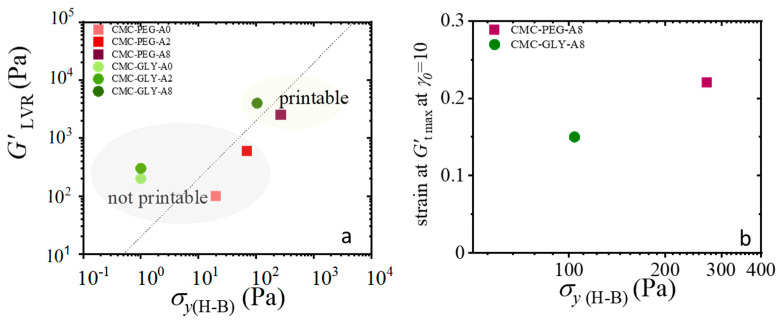
(**a**) Relationship between the storage modulus in the linear viscoelastic regime (*G′_LVR_*) and the yield stress calculated from steady-shear flow (*σ*_*y*(H–B)_). (**b**) Relationship between the elastic strain at the point of maximum elasticity (*G′_tmax_*) and the yield stress calculated from steady-shear flow (*σ*_*y*(H–B)_) for an applied strain amplitude of 10 strain units.

The storage modulus, obtained from small-amplitude oscillatory shear under linear viscoelastic conditions (*G′*_LVR_), and the yield stress, calculated from steady shear flow curves fitted to Herschel–Bulkley model (*σ*_*y*(H-B)_), served as key indicators of the printability of these hydrogels. Figure 11a compiles these parameters into an Ashby-type diagram that visually represents the strength and flowability of the hydrogels.

The evaluation of the yield-stress parameter in this context also aligns with the observed physical processes during oscillatory yielding, as demonstrated in Figure 10a,b. This suggests that the evaluation of yield stress using different methodologies can provide insights into the hydrogel’s flow behavior, directly affecting its ability to print with precision. Interestingly, the main difference in the printability between CMC-PEG-A8 and CMC-GLY-A8 hydrogels was found to be linked to their ability to acquire recoverable strain during the yielding process. The ability of the hydrogel to recover its shape after deformation (i.e., its recoverability) is a critical factor influencing printability. As shown in Figure 8, CMC-PEG-A8 hydrogels with better recoverability are likely to retain their shape and structural integrity more effectively during and after the printing process, leading to the observed higher resolution and better-defined printed structures. Therefore, to summarize these results, Figure 11b highlights that the CMC-PEG-A8 hydrogel, despite having a lower *G′_LVR_* modulus, has both a higher recoverable strain under flow conditions and a higher yield stress. These findings underscore the importance of balancing rheological properties like yield stress, storage modulus, and recoverability to optimize the printability and performance of hydrogels for 3D-printing applications.

## 4. Materials and Methods

### 4.1. CMC-Based Hydrogels Formulations

Two different types of hydrogels were prepared: (1) formulations prepared with CMC and polyethylene glycol, CMC-PEG; and (2) formulations prepared with CMC and glycerol, CMC-GLY. The two CMC hydrogels were used to encapsulate the drug atenolol, with concentrations varying between 0, 2, and 8 wt%. The specific formulation is presented in the Table 3.

The CMC-PEG and CMC-GLY formulations were adjusted to achieve similar hydrogel strength (G′ value) for the reference formulations without atenolol, as discussed in the Results section.

For the PEG-based formulations, PEG was first added to a beaker. The PEG was heated to 85 °C to facilitate melting, and when it became transparent, the CMC and atenolol were added. A magnetic stirrer was used for mixing. Once a uniform dispersion of the ingredients was achieved, the beaker was removed from the hotplate, and the required amount of water was added in a single step. The mixture was then stirred until a homogeneous solution was obtained. In the case of the glycerol-based hydrogels, the application of heat was not required, although the methodology was otherwise similar.

All formulations were tested at 7 days post-fabrication, as prior tests confirmed the stability of the hydrogel network.

**Figure 12:**
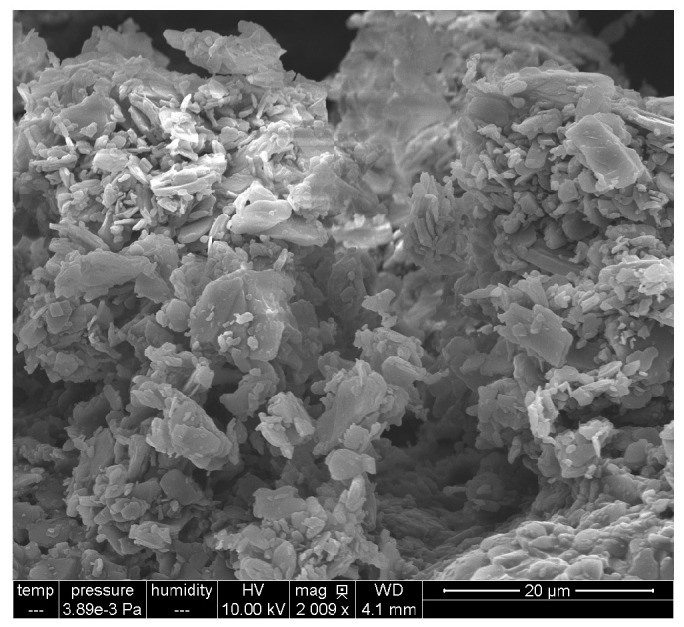
SEM picture of the lyophilized CMC-GLY-A8 hydrogel.

The crystalline atenolol particles characterized by SEM in the lyophilized CMC-GLY–A8 hydrogel are shown in Figure 12.

All of the formulation ingredients were purchased from Sigma-Aldrich: carboxymethyl cellulose sodium (9004-32-4), polyethylene glycol (Mn = 6000, 901397), glycerol (56-81-5), and atenolol (29122-68-7).

### 4.2. Three-Dimensional Printing

The Envisiontec 3D-Bioplotter^®^ Developer series (Gladbeck, Germany) was used to fabricate the oral forms. Luer lock needle tips (inner diameter of 0.4 mm) were used at room temperature, under different pressures and printing speeds, achieving 3D construction with relatively good accuracy.

**Figure 13:**
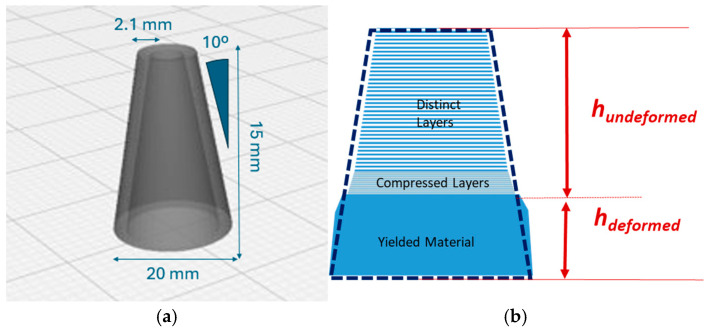
(**a**) Geometry of the miniaturized slump cone designed to calculate the printed strength of our CMC-based hydrogels. (**b**) Diagram showing the yielding during printing: the upper layers are distinct and uncompressed, the layers below the distinct layers are compressed, and the bottom ones are yielded. The height of the non-yielded part, *h_undeformed_*, allows us to calculate the yield strength of the printed object. (Adapted with permission of Reference [7].)

A miniaturized slump cone (DIW slump cone) with similar dimensions to that reported by Liu et al. [7] was used to evaluate the shape consistency of our hydrogel-printed objects (Figure 13a). The cone was designed to keep the original wall angle (θ = 10°) of the ASTM C143 standard [53] but features a reduced base diameter (20 mm) and a thinner wall (2.1 mm, formed by seven 0.3 mm thick lines).

The ability of the printed slump cone to retain its shape serves as a metric of material strength in DIW. As the material is printed, gravity-induced deformation provides insights into its yield properties. As reported by Liu et al. [7], the printed material can be described to contain three regions (illustrated in Figure 13b): the top region, where layers remain distinct and undeformed; the middle region, where layers experience compression; and the bottom region where the material yields and bulges, deviating from the initial 10° angle.

A total of 3 DIW slump cones each were printed for CMC-GLY-A8 and CMC-PEG-A8 formulations. The DIW slump cones were printed at a print speed of 30 mm/s, with a total printing height of 15 mm. Measurements of the undeformed height were performed immediately after completion of the slump cone.

### 4.3. Rheological Measurements

The rheological study was carried out using a strain-controlled rheometer, ARES G2 TA instruments (New Castle, DE, USA). All measurements were carried out using a 1 mm gap and 25 mm parallel plate with a solvent trap. The temperature was maintained at 25 °C using a Peltier plate.

The steady-shear flow curves were determined using two procedures with different shear rate ranges. The first one covers a shear rate range of $\dot{\gamma }$ = 0.001–1 s^−1^, and the second one extends from 0.01 s^−1^ to the highest shear rates, up to 100 s^−1^.

LAOS measurements were carried out in the form of strain amplitude sweep tests: an oscillatory input strain was applied, and the correspondent output stress was registered for every strain amplitude. The strain amplitude between 0.1% and 1000% were chosen to investigate the structure deformation from small amplitude oscillatory at a constant frequency of 1 rad/s. Stress–strain close loops were generated for 12 oscillatory cycles. For each formulation, at least two replicates were tested in each measurement. The curves were revised, and only cycles meeting the alternance steady-state condition were analyzed in this work.

The obtained Lissajous–Bowditch curves were analyzed using (1) FTR and FTR-SD methodology using the TRIOS^®^ v.5.1^.^-software, TA instruments (New Castle, DE, USA).; and (2) SPP freely available data analysis package, kindly provided by Prof. Simon A. Rogers (University of Illinois, Champaign IL (EEUU)).

#### 4.3.1. Fourier-Transform Rheology, FTR

The transition from linear to nonlinear behavior of hydrogels was evaluated using the FTR procedure, which was well established by Wilhelm et al. [26] and Ewoldt et al. [37]. Strain sweep tests at a constant frequency of 1 rad/s were performed. After Fourier analysis, the nonlinear shear stress can be expressed by Equation (8) using the intensities, $I_{n}$, and phases, $\delta_{n}$, of the integer number. $I_{n}$ and $\delta_{n}$ were evaluated from the intensity and phase spectra, I (ω) and δ (ω) (Equations (9) and (10)), at integer multiples of the applied fundamental frequency, ω_1_ [26]:

(8) $$\sigma \left(t\right)=\sum_{n\epsilon N}I_{n}\left(\sin\left(n\omega_{1}t\right)+\delta_{n}\right)$$

(9) $$I_{n}=I(n\omega_{1})$$

(10) $$\delta_{n}=\delta \left(n\omega_{1}\right)$$

In addition to the analysis of the fundamental and odd harmonics, for every n harmonic, an intrinsic nonlinear parameter, ^n^Q (γ_0_, ω), can be defined in the limit of small-strain amplitudes, ^n^Q_0_ (ω). The parameter, which is only frequency-dependent, is defined through the following equation [26]:

(11) Q0nω=limγ0→0⁡Qn γ0,ωwith Qn γ0,ω=In/1 γ0n−1

^n^Q_0_ (ω) provides information on the inherent nonlinear material properties of a sample, as the trivial scaling, $I_{n/1}\propto \;\gamma_{0}^{n-1}$, is eliminated. In the MAOS region, with the presence of the third harmonic with the highest intensity, n = 3, we used the following equations:

(12) $$Q\equiv \frac{I_{3/1}}{\gamma_{0}^{2}}$$

(13) $$\underset{\gamma \to 0}{\lim}Q\equiv Q_{0}$$

#### 4.3.2. Fourier-Transform–Stress-Decomposition Rheology, FTR-SD

Similar to the linear viscoelastic approach under small-amplitude oscillatory shear (SAOS) conditions, the original nonlinear stress wave, *σ*(t), can also be reconstructed from the elastic modulus, *G′*, and the viscous modulus, *G″*. The elastic stress, *σ′*(t), and viscous stress, *σ″*(t), are expressed by Equations (14) and (15), following the stress decomposition (SD) method in terms of the Chebyshev coefficients (e_n_ and v_n_) [35,37].

(14) $$\sigma^{\prime }\left(t\right)=\frac{\sigma \left(\gamma ,\dot{\gamma }\right)-\sigma \left(-\gamma ,\dot{\gamma }\right)}{2}=\gamma_{0}\sum_{n,odd}G_{n}^{\prime }\sin\left(n\omega t\right)$$

(15) $$\sigma^{\prime \prime }\left(t\right)=\frac{\sigma \left(\gamma ,\dot{\gamma }\right)-\sigma \left(\gamma ,-\dot{\gamma }\right)}{2}=\gamma_{0}\sum_{n,odd}G_{n}^{\prime \prime }\cos\left(n\omega t\right)$$

The relationships of the Fourier coefficients in the time domain and the Chebyshev coefficients in the strain or strain-rate domain are given by

$$e_{n}=G_{n}^{\prime }{\left(-1\right)}^{\frac{n-1}{2}},\;n:odd,$$

(16) $$\nu_{n}=\frac{G_{n}^{\prime \prime }}{\omega }=\eta_{n}^{\prime }\;n:odd.\;$$

The first-order Chebyshev coefficients ($e_{1}$ and $\nu_{1}$) define the viscoelastic properties in the linear region (i.e., $e_{1}=G\prime$ and $\nu_{1}=G^{\prime \prime }/\omega )$. Any deviation from linearity (i.e., the n = 3 harmonic) is interpreted depending on the sign of $e_{3}\;$ and $\nu_{3}$. A positive third-order contribution results in higher elastic (or viscous) stress at maximum strain (or strain rate) that is represented by the first-order contribution alone. Thus, depending on the sign of the third-order coefficient, the following physical interpretation is suggested:

(17) $$e_{3}=-G_{3}^{\prime }\left\{\begin{array}{c} >0\;strain-stiffening\\ =0\;linear\;elastic\\ <0\;strain-softening \end{array}\;\;\;\;\;\;\;\;\;\;\;\;\;\;\;\;\;\right.\nu_{3}=-\frac{G_{3}^{\prime \prime }}{\omega }\left\{\begin{array}{c} >0\;shear-thickening\\ =0\;linear\;viscous\\ <0\;shear-thinning \end{array}\right.$$

Using their Chebyshev coefficients, Ewoldt et al. [38] also defined novel local viscoelastic moduli called the large-strain modulus ($G_{L}^{\prime }$) and minimum-strain modulus ($G_{M}^{\prime }$), and the minimum-rate dynamic viscosity ($\eta_{M}^{\prime }$) and large-rate dynamic viscosity ($\eta_{L}^{\prime }$). These intra-cycle nonlinearities are defined in terms of either differential or secant measures at maximum and minimum strains and rates by the following:

(18) $$G_{M}^{\prime }\equiv \;{\left.\frac{d\sigma \;}{d\gamma }\right]}_{\gamma =0}=\sum nG_{n}^{\prime }=e_{1}-3e_{3}+5e_{5}-7e_{7}+\cdots$$

(19) $$G_{L}^{\prime }\equiv \;{\left.\frac{\sigma \;}{\gamma }\right]}_{\gamma =\pm \gamma_{0}}=\sum G_{n}^{\prime }\;{\left(-1\right)}^{\frac{\left(n-1\right)}{2}}=e_{1}+e_{3}+e_{5}+e_{7}+\cdots$$

(20) $$\eta_{M}^{\prime }\equiv \;{\left.\frac{d\sigma \;}{d\dot{\gamma }}\right]}_{\dot{\gamma }=0}=\frac{1}{\omega }\sum nG_{n}^{\prime \prime }\;{\left(-1\right)}^{\frac{\left(n-1\right)}{2}}=\nu_{1}-3\;\nu_{3}+5\nu_{5}-7e_{7}+\cdots$$

(21) $$\eta_{L}^{\prime }\equiv \;{\left.\frac{\sigma \;}{\dot{\gamma }}\right]}_{\dot{\gamma }=\pm {\dot{\gamma }}_{0}}=\frac{1}{\omega }\sum G_{n}^{\prime \prime }=\nu_{1}+\nu_{3}+\nu_{5}+e_{7}+\cdots \;\;$$

$G_{M}^{\prime }$ and $G_{L}^{\prime }$ converged to the linear elastic modulus in the LVR region. These local measures were then combined to develop dimensionless indices intended to quantifiy a relative amount of strain stiffening (S) and shear thickening (T).

(22) $$S\equiv \frac{G_{L}^{\prime }-G_{M}^{\prime }}{G_{L}^{\prime }}$$

(23) $$T\equiv \frac{\eta_{L}^{\prime }-\eta_{M}^{\prime }}{\eta_{L}^{\prime }}$$

Therefore, equivalent to the use of the Chebyshev coefficient in Equation (17), the intra-cycle elastic and viscous nonlinearity response to LAOS deformation can be interpreted in terms of the dimensionless indices, S and T, as follows:

(24) $$S\;\left\{\begin{array}{c} >0\;strain-stiffening\\ =0\;linear\;elastic\\ <0\;strain-softening \end{array}\;\;\;\;\;\;\;\;\;\;\;\;\;\;\;\;\;\right.T\left\{\begin{array}{c} >0\;shear-thickening\\ =0\;linear\;viscous\\ <0\;shear-thinning \end{array}\right.$$

#### 4.3.3. Sequence of Physical Processes Analysis, SPP

The SPP framework evaluates the rheological response of a material from the time evolution of the values of strain, γ(t); strain rate, $\dot{\gamma }(t)$; and stress, *σ*(t). For this purpose, the SPP methodology considers each point of an oscillation cycle to be given by a position vector, P(t) = [γ (t), $\dot{\gamma }\left(t\right)$, σ(t)]. The mathematical description of P(t) is defined by orthonormal vectors, namely normal, N(t); tangent, T(t); and binormal, B(t), vectors, according to the Frenet–Serret theorem [38,39]. The tangent vector, T(t), points to the direction of flow, which is tangent to the L-B curve defined by the time derivative of the position vector, T(t) = P′(t)/|P′(t)|. The normal vector, N(t), points to the center of the curvature defined by the time derivative of the tangent vector, N(t) = T′(t)/|T′(t)|. These two vectors, T(t) and N(t), span the osculating plane of the deformation, while the third binormal vector, B(t), is orthonormal to both T(t) and N(t) vectors: $\vec{B(t)}=\vec{T(t)}\times \vec{N(t)}$ [38,39].

The projections of the binormal vector, $B\left(t\right)=(B_{\gamma },B_{\dot{\gamma }/\omega },B_{\sigma })$, and the orientation of the osculating plane along the strain, strain rate, and stress curve define the instantaneous-transient elastic, $G_{t}^{\prime }\left(t\right)$, and viscous, $G_{t}^{\prime \prime }\left(t\right)$, moduli.

(25) $$G_{t}^{\prime }=-\frac{B_{\gamma }}{B_{\sigma }}=\frac{d\sigma }{d\gamma }$$

(26) $$G_{t}^{\prime \prime }=-\frac{B_{\dot{\gamma }/\omega }}{B_{\sigma }}=\frac{d\sigma }{d(\dot{\gamma }/\omega )}$$

The raw waveform data, i.e., σ(t), γ(t), and $\dot{\gamma }$(t), were analyzed using the SPP freeware for MATLAB R2023b provided by its developers (https://publish.illinois.edu/rogerssoftmatter/freeware/), with access on 1 September 2022. The SPP analysis was performed in a full cycle, using Fourier domain filtering, where the first 19 harmonics are used to reconstruct the waveform.

## Figures and Tables

**Table 1:** Rheological parameters of the Herschel–Bulkley model.

CMC-Based Formulation	Yield Stress, $\sigma_{y(H-B)}$ (Pa)	Consistency Index, $k(\mathrm{Pa}\cdot s^{n})$	Flow Index, n	*R* ^2^
CMC-PEG-A0	17.98	17.60	0.69	0.9856
CMC-PEG-A2	66.24	39.55	0.51	0.9972
CMC-PEG-A8	275	279	0.85	0.9904
CMC-GLY-A0	0	76.35	0.74	0.9999
CMC-GLY-A2	0.36	78.19	0.70	0.9998
CMC-GLY-A8	104.78	525.43	0.43	0.9875

**Table 2:** Yield stress calculated using different approaches: printing DIW cone slump, steady-shear flow, and large amplitude oscillatory shear LAOS.

Yield Stress (Pa)	CMC-PEG-A8	CMC-GLY-A8	Rheological Methodology
$\sigma_{y}^{p}\;$ DIW cone slump	>147	80	Printing strength (Equation (1))
$\sigma_{y}$_(H-B)_ Steady-shear flow	275	104	Herschel–Bulkley model (Equation (2))
$\sigma_{y}$_(H-B)o_ L-B plot with $\gamma_{0}$ = 10 strain units	274	227	Herschel–Bulkley model (Equation (2))
$\sigma_{y\;}$(G′ = G″) Oscillatory strain sweep at ω = 1 rad/s	549	434	Viscoelastic flow condition G′ = G″
$\sigma_{y\;}$ at *G′_tmax_* Cole–Cole plot at $\gamma_{0}$ = 10 strain units	504	405	SPP analysis Stress at the maximum elasticity

**Table 3:** Specific formulation for the development of CMC-PEG and CMC-GLY hydrogels loaded with atenolol.

Formulation (wt%)	PEG-A0	PEG-A2	PEG-A8	GLY-A0	GLY-A2	GLY-A8
Atenolol	-	2	8	-	2	8
CMC	5	5	5	12.5	12.5	12.5
PEG6000	20	20	20			
water	75	73	67	5	5	5
Glycerol	-	-	-	82.5	80.5	74.5

## Data Availability

The original contributions presented in this study are included in the article. Further inquiries can be directed to the corresponding authors.

## References

[1] Soleymani S., Naghib S.M. (2023). 3D and 4D printing hydroxyapatite-based scaffolds for bone tissue engineering and regeneration. Heliyon.

[2] Honkamäki L., Kulta O., Puistola P., Hopia K., Emeh P., Isosaari L., Mörö A., Narkilahti S. (2024). Hyaluronic Acid-Based 3D Bioprinted Hydrogel Structure for Directed Axonal Guidance and Modeling Innervation In Vitro. Adv. Healthc. Mater..

[3] Qiu B., Bessler N., Figler K., Buchholz M.B., Rios A.C., Malda J., Levato R., Caiazzo M. (2020). Bioprinting Neural Systems to Model Central Nervous System Diseases. Adv. Funct. Mater..

[4] Baniasadi H., Abidnejad R., Fazeli M., Lipponen J., Niskanen J., Kontturi E., Seppälä J., Rojas O.J. (2024). Innovations in hydrogel-based manufacturing: A comprehensive review of direct ink writing technique for biomedical applications. Adv. Colloid Interface.

[5] Chen J.X.M., Chen T., Zhang Y., Fang W., Li W.E., Li T., Popovic M.R., Naguib H.E. (2024). Conductive Bio-based Hydrogel for Wearable Electrodes via Direct Ink Writing on Skin. Adv. Funct. Mater..

[6] Jiang P., Yan C., Guo Y., Zhang X., Cai M., Jia X., Wang X., Zhou F. (2019). Direct ink writing with high-strength and swelling-resistant biocompatible physically crosslinked hydrogels. Biomater. Sci..

[7] Liu Y., Hildner M., Roy O., Bogert W.A.V.D., Lorenz J., Desroches M., Koppi K., Shih A., Larson R.G. (2023). On the selection of rheological tests for the prediction of 3D printability. J. Rheol..

[8] Wei P., Cipriani C., Hsieh C.M., Kamani K., Rogers S., Pentzer E. (2023). Go with the flow: Rheological requirements for direct ink write printability. J. Appl. Phys..

[9] Rau D.A., Bortner M.J., Williams C.B. (2023). A rheology roadmap for evaluating the printability of material extrusion inks. Addit. Manuf..

[10] Rau D.A., Williams C.B., Bortner M.J. (2023). Rheology and printability: A survey of critical relationships for direct ink write materials design. Prog. Mater. Sci..

[11] Agrawal R., García-Tuñón E. (2024). Interplay between yielding, “recovery”, and strength of yield stress fluids for direct ink writing: New insights from oscillatory rheology. Soft Matter.

[12] García-Tuñón E., Agrawal R., Ling B., Dennis D.J.C. (2023). Fourier-transform rheology and printability maps of complex fluids for three-dimensional printing. Phys. Fluids.

[13] Calafel I., Aguirresarobe R.H., Peñas M.I., Santamaria A., Tierno M., Conde J.I., Pascual B. (2020). Searching for rheological conditions for FFF 3D printing with PVC based flexible compounds. Materials.

[14] Corker A., Ng H.C.H., Poole R.J., García-Tuñón E. (2019). 3D printing with 2D colloids: Designing rheology protocols to predict “printability” of soft-materials. Soft Matter.

[15] Capitani D., Del Nobile M.A., Mensitieri G., Sannino A., Segre A.L. (2000). 13C solid-state NMR determination of cross-linking degree in superabsorbing cellulose-based networks. Macromolecules.

[16] Mallakpour S., Tukhani M., Hussain C.M. (2021). Recent advancements in 3D bioprinting technology of carboxymethyl cellulose-based hydrogels: Utilization in tissue engineering. Adv. Colloid Interface Sci..

[17] Coussot P., Rogers S.A. (2021). Oldroyd’s model and the foundation of modern rheology of yield stress fluids. J. Non-Newton. Fluid. Mech..

[18] Kamani K., Donley G.J., Rogers S.A. (2021). Unification of the Rheological Physics of Yield Stress Fluids. Phys. Rev. Lett..

[19] Erturk M.Y., Rogers S.A., Kokini J. (2022). Comparison of Sequence of Physical Processes (SPP) and Fourier Transform Coupled with Chebyshev Polynomials (FTC) methods to Interpret Large Amplitude Oscillatory Shear (LAOS) Response of Viscoelastic Doughs and Viscous Pectin Solution. Food Hydrocoll..

[20] Rogers S.A., Erwin B.M., Vlassopoulos D., Cloitre M. (2011). A sequence of physical processes determined and quantified in LAOS: Application to a yield stress fluid. J. Rheol..

[21] Donley G.J., Singh P.K., Shetty A., Rogers S.A., Weitz D.A. (2020). Elucidating the G″ overshoot in soft materials with a yield transition via a time-resolved experimental strain decomposition. Proc. Natl. Acad. Sci. USA.

[22] Kamani K.M., Donley G.J., Rao R., Grillet A.M., Roberts C., Shetty A., Rogers S.A. (2023). Understanding the transient large amplitude oscillatory shear behavior of yield stress fluids. J. Rheol..

[23] Rogers S. (2018). Large amplitude oscillatory shear: Simple to describe, hard to interpret. Phys. Today.

[24] Rogers S.A., Park J.D., Lee C.W.J. (2019). Instantaneous dimensionless numbers for transient nonlinear rheology. Rheol. Acta.

[25] Hyun K., Wilhelm M., Klein C.O., Cho K.S., Nam J.G., Ahn K.H., Lee S.J., Ewoldt R.H., McKinley G.H. (2011). A review of nonlinear oscillatory shear tests: Analysis and application of large amplitude oscillatory shear (LAOS). Prog. Polym. Sci..

[26] Wilhelm M. (2002). Fourier-transform rheology. Macromol. Mater. Eng..

[27] Vittorias I., Wilhelm M. (2007). Application of FT rheology to industrial linear and branched polyethylene blends. Macromol. Mater. Eng..

[28] Wilhelm M., Reinheimer P., Ortseifer M. (1999). High sensitivity Fourier-transform rheology. Rheol. Acta.

[29] Hassanabadi H.M., Wilhelm M., Rodrigue D. (2014). A rheological criterion to determine the percolation threshold in polymer nano-composites. Rheol. Acta.

[30] Reinheimer K., Grosso M., Hetzel F., Kübel J., Wilhelm M. (2012). Fourier Transform Rheology as an innovative morphological characterization technique for the emulsion volume average radius and its distribution. J. Colloid Interface Sci..

[31] Ahirwal D., Filipe S., Neuhaus I., Busch M., Schlatter G., Wilhelm M. (2014). Large amplitude oscillatory shear and uniaxial extensional rheology of blends from linear and long-chain branched polyethylene and polypropylene. J. Rheol..

[32] Kempf M., Ahirwal D., Cziep M., Wilhelm M. (2013). Synthesis and linear and nonlinear melt rheology of well-defined comb architectures of PS and PpMS with a low and controlled degree of long-chain branching. Macromolecules.

[33] Hoyle D.M., Auhl D., Harlen O.G., Barroso V.C., Wilhelm M., McLeish T.C.B. (2014). Large amplitude oscillatory shear and Fourier transform rheology analysis of branched polymer melts. J. Rheol..

[34] Song H.Y., Faust L., Son J., Kim M., Park S.J., Ahn S.-K., Wilhelm M., Hyun K. (2020). Small and medium amplitude oscillatory shear rheology of model branched polystyrene (PS) melts. Polymers.

[35] Cho K.S., Hyun K., Ahn K.H., Lee S.J. (2005). A geometrical interpretation of large amplitude oscillatory shear response. J. Rheol..

[36] Dimitriou C.J., Ewoldt R.H., McKinley G.H. (2013). Describing and prescribing the constitutive response of yield stress fluids using large amplitude oscillatory shear stress (LAOStress). J. Rheol..

[37] Ewoldt R.H., Hosoi A.E., McKinley G.H. (2008). New measures for characterizing nonlinear viscoelasticity in large amplitude oscillatory shear. J. Rheol..

[38] Lee C.W., Rogers S.A. (2017). A sequence of physical processes quantified in LAOS by continuous local measures. Korea Aust. Rheol. J..

[39] Rogers S.A., Lettinga M.P. (2012). A sequence of physical processes determined and quantified in large-amplitude oscillatory shear (LAOS): Application to theoretical nonlinear models. J. Rheol..

[40] Park J.D., Rogers S.A. (2020). Rheological manifestation of microstructural change of colloidal gel under oscillatory shear flow. Phys. Fluids.

[41] Rogers S.A. (2017). In search of physical meaning: Defining transient parameters for nonlinear viscoelasticity. Rheol. Acta.

[42] Shim Y.H., Griebler J.J., Rogers S.A. (2024). A reexamination of the Cox–Merz rule through the lens of recovery rheology. J. Rheol..

[43] Kim Y., Kim S., Kim B.S., Park J.H., Ahn K.H., Park J.D. (2022). Yielding behavior of concentrated lithium-ion battery anode slurry. Phys. Fluids.

[44] Song H.Y., Kim S.Y., Park M.S., Park J.D., Hyun K. (2024). Effect of stirring time on viscoelastic properties of liquid gallium-oxide amalgams. Korea Aust. Rheol. J..

[45] Ulbricht J., Jordan R., Luxenhofer R. (2014). On the biodegradability of polyethylene glycol, polypeptoids and poly(2-oxazoline)s. Biomaterials.

[46] Safaei H.R., Shekouhy M., Rahmanpur S., Shirinfeshan A. (2012). Glycerol as a biodegradable and reusable promoting medium for the catalyst-free one-pot three component synthesis of 4H-pyrans. Green Chem..

[47] Patel D., Doshi D.H., Desai A. (1997). Short-term stability of atenolol in oral liquid formulations. Int. J. Pharm. Compd..

[48] Laracuente M.-L., Yu M.H., McHugh K.J. (2020). Zero-order drug delivery: State of the art and future prospects. J. Control. Release.

[49] Hossieni-Aghdam S.J., Foroughi-Nia B., Zare-Akbari Z., Mojarad-Jabali S., Motasadizadeh H., Farhadnejad H. (2018). Facile fabrication and characterization of a novel oral pH-sensitive drug delivery system based on CMC hydrogel and HNT-AT nanohybrid. Int. J. Biol. Macromol..

[50] Adhikari S.N.R., Nayak B.S., Nayak A.K., Mohanty B. (2010). Formulation and Evaluation of Buccal Patches for Delivery of Atenolol. AAPS Pharmscitech.

[51] Cassano R., Sole R., Siciliano C., Baldino N., Mileti O., Procopio D., Curcio F., Calviello G., Serini S., Trombino S. (2024). Eutectogel-Based Drug Delivery: An Innovative Approach for Atenolol Administration. Pharmaceutics.

[52] Preeti, Sambhakar S., Malik R., Bhatia S., Al Harrasi A., Saharan R., Aggarwal G., Kumar S., Sehrawat R., Rani C. (2024). Lipid Horizons: Recent Advances and Future Prospects in LBDDS for Oral Administration of Antihypertensive Agents. Int. J. Hypertens..

[53] (2020). Test Method for Slump of Hydraulic-Cement Concrete.

[54] Herrada-Manchón H., Rodríguez-González D., Fernández M.A., Kucko N.W., Groot F.B.-D., Aguilar E. (2022). Effect on Rheological Properties and 3D Printability of Biphasic Calcium Phosphate Microporous Particles in Hydrocolloid-Based Hydrogels. Gels.

[55] Mueller S., Llewellin E.W., Mader H.M. (2010). The rheology of suspensions of solid particles. Proc. R. Soc. A Math. Phys. Eng. Sci..

[56] Nelson A.Z., Ewoldt R.H. (2017). Design of yield-stress fluids: A rheology-to-structure inverse problem. Soft Matter.

[57] Doraiswamy D., Mujumdar A.N., Tsao I., Beris A.N., Danforth S.C., Metzner A.B. (1991). The Cox–Merz rule extended: A rheological model for concentrated suspensions and other materials with a yield stress. J. Rheol..

[58] Merger D. (2015). Large Amplitude Oscillatory Shear Investigations of Colloidal Systems: Experiments and Constitutive Model Predictions. Doctoral Dissertation.

[59] Hyun K., Kim W. (2011). A new non-linear parameter Q from FT-Rheology under nonlinear dynamic oscillatory shear for polymer melts system. Korea Aust. Rheol. J..

[60] Sangroniz L., Palacios J.K., Fernández M., Eguiazabal J.I., Santamaria A., Müller A.J. (2016). Linear and non-linear rheological behavior of polypropylene/polyamide blends modified with a compatibilizer agent and nanosilica and its relationship with the morphology. Eur. Polym. J..

[61] Sandoval A.J., Fernández M., Sanz O., Santamaría A., Penott-Chang E., Müller A.J. (2022). Large amplitude oscillatory shear (LAOS) behavior of chocolates of different compositions. J. Rheol..

[62] Leblanc J.L. (2005). Investigating the Non-Linear Viscoelastic Behavior of Filled Rubber Compounds Through Fourier Transform Rheometry. Rubber Chem. Technol..

[63] Heymann L., Peukert S., Aksel N. (2002). Investigation of the solid–liquid transition of highly concentrated suspensions in oscillatory amplitude sweeps. J. Rheol..

[64] Kallus S., Willenbacher N., Kirsch S., Distler D., Neidhöfer T., Wilhelm M., Spiess H.W. (2001). Characterization of polymer dispersions by Fourier transform rheology. Rheol. Acta.

[65] Hyun K., Nam J.G., Wilhellm M., Ahn K.H., Lee S.J. (2006). Large amplitude oscillatory shear behavior of PEO-PPO-PEO triblock copolymer solutions. Rheol. Acta.

[66] Shi J., Rogers S.A. (2023). The benefits of a formalism built on recovery: Theory, experiments, and modeling. J. Non-Newton. Fluid. Mech..

[67] Park J.D., Rogers S.A. (2018). The transient behavior of soft glassy materials far from equilibrium. J. Rheol..

[68] Lee J.C.W., Porcar L., Rogers S.A. (2019). Unveiling temporal nonlinear structure-rheology relationships under dynamic shearing. Polymers.

[69] van der Vaart K., Rahmani Y., Zargar R., Hu Z., Bonn D., Schall P. (2013). Rheology of concentrated soft and hard-sphere suspensions. J. Rheol..

[70] Radhakrishnan R., Fielding S.M. (2018). Shear banding in large amplitude oscillatory shear (LAOStrain and LAOStress) of soft glassy materials. J. Rheol..

[71] Poulos A.S., Stellbrink J., Petekidis G. (2013). Flow of concentrated solutions of starlike micelles under large-amplitude oscillatory shear. Rheol. Acta.

